# Non-redundant roles of the phosphoinositide phosphatases PTEN and PIPP in PI3K/AKT signaling in breast cancer

**DOI:** 10.1038/s42003-025-09364-2

**Published:** 2025-12-17

**Authors:** Lisa M. Ooms, Daniel T. Ferguson, Samuel J. Rodgers, Karmanpreet K. Sukhija, Emily I. Jones, Mariah P. Csolle, Hon Yan Kelvin Yip, Roger J. Daly, Tony Tiganis, Catriona A. McLean, Antonella Papa, Christina A. Mitchell

**Affiliations:** 1 Cancer Program, Monash Biomedicine Discovery Institute and Department of Biochemistry and Molecular Biology, Monash University, Clayton, VIC Australia; 2 Department of Anatomical Pathology, Alfred Hospital, Prahran, VIC Australia; 3 Present Address: Centre for Cancer Cell Reprogramming, Faculty of Medicine, University of Oslo, Oslo, Norway, and Department of Molecular Cell Biology, Institute for Cancer Research, Oslo University Hospital, Oslo, Norway; 4 Present Address: South Australian immunoGENomics Cancer Institute (SAiGENCI), University of Adelaide, Adelaide, SA Australia

**Keywords:** Breast cancer, Phosphoinositol signalling

## Abstract

Phosphoinositide 3-kinase (PI3K) signaling is hyperactivated in ~70% of breast cancers via mutations in oncogenes including *PIK3CA* or inactivation/depletion of phosphoinositide (PI)-phosphatases. Generation of PI(3,4,5)P_3_ by PI3K activates many downstream effectors, including AKT, that induce cellular proliferation in breast cancer. In this context PI(3,4,5)P_3_ is tightly regulated by PI-phosphatases, including the tumor suppressor PTEN and inositol polyphosphate 5-phosphatases such as PIPP/INPP5J. PTEN and PIPP dephosphorylate PI(3,4,5)P_3_ to form different lipid products, thereby individually regulating AKT activation. PI3K/AKT signaling is complex and the functional interplay between these PI-phosphatases in suppressing this pathway in vivo is unknown. Here, we utilize experimental models of breast cancer, both dependent and independent of *PIK3CA* mutation. *Pipp* ablation in *Pten*^+/−^ mice increases mammary AKT signaling and cell proliferation, associated with increased hyperplasia and ductal thickening, characteristics linked with mammary epithelial cell transformation. In breast cancer cell lines, combined *PIPP*/*PTEN* knockdown increases AKT signaling and cell proliferation, independent of mutant *PIK3CA*, above any single PI-phosphatase knockdown. Notably, combined *PIPP/PTEN* loss is observed in a subset of human breast cancers, associated with reduced survival. Collectively, these findings support a model whereby loss of *PIPP* constitutes a co-operative step towards breast cancer progression in the context of *PTEN* deficiency.

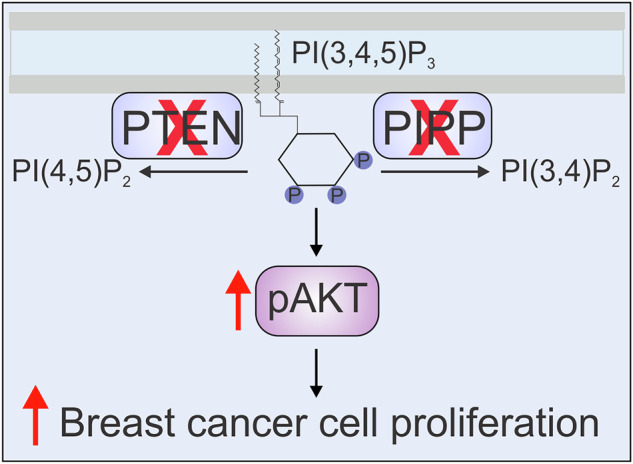

## Abstract (web summary)

Experimental models of breast cancer reveal the phosphoinositide-phosphatases PIPP and PTEN play non-redundant roles in suppressing mouse mammary epithelial cell transformation, and disease progression in a subset of human breast cancers.

## Introduction

The class I phosphoinositide 3-kinase (PI3K) signaling pathway stimulates a complex network that facilitates cell proliferation and cancer progression. Growth factors activate class I PI3Ks to phosphorylate the inositol head group of the membrane-bound lipid, PI(4,5)P_2_ to form PI(3,4,5)P_3_, which in turn promotes the translocation and/or activation of a variety of PH domain-containing proteins such as the serine/threonine protein kinases PDK1 and AKT1/2/3^1,2^. PI3K/AKT signaling is hyperactivated in ~70% of human breast cancers, associated with disease progression and therapy resistance^3^. Alteration of this pathway occurs via mutations in oncogenes, such as the catalytic subunit of the class I PI3K (*PIK3CA*) or *AKT1*, in ~40% and ~7% of estrogen receptor positive (ER+) breast cancers, respectively^4–7^. Alternatively, in this context, the inactivation of tumor suppressors such as phosphatase and tensin homolog deleted on chromosome 10 (PTEN) or depletion of the inositol polyphosphate 4-phosphatase type II (INPP4B) also leads to hyperactivated PI3K/AKT signaling^8–10^.

PI(3,4,5)P_3_ is hydrolyzed by various PI-phosphatases producing distinct phosphoinositide species. PTEN hydrolyzes the 3-phosphate group from PI(3,4,5)P_3_ to produce PI(4,5)P_2_ which prevents AKT activation, but induces actin cytoskeleton reorganization, vesicular trafficking and membrane permeability. By contrast, the inositol polyphosphate 5-phosphatases hydrolyze the 5-phosphate group from PI(3,4,5)P_3_ to form PI(3,4)P_2_ which binds AKT with similar affinity to PI(3,4,5)P_3_. PI(3,4)P_2_ facilitates endocytosis, lamellipodia, and podosome/invadopodia turnover^8,11,12^. PI(3,4)P_2_ can be further hydrolyzed by PTEN and/or INPP4A/B to form PI(4)P or PI(3)P, respectively, to terminate PI3K/AKT signaling^13^.

Germline heterozygous mutation of *PTEN* in humans gives rise to PTEN-hamartoma syndromes, such as Cowden Syndrome (CS), associated with the development of multiple hamartomas and increased lifetime risk of breast and thyroid cancers^14,15^. In sporadic breast cancer, monoallelic loss of *PTEN* occurs in 30–40% of cases and biallelic loss in 5%^16^. Despite the relative rarity of biallelic loss, absence of the PTEN protein is observed in ~30% of breast cancers due to promoter methylation, loss of heterozygosity, protein instability or post-transcriptional mechanisms^17^. Loss of *PTEN* correlates with breast cancer progression, poor prognosis and reduced response to targeted therapies^17–21^.

The inositol polyphosphate 5-phosphatases comprise ten mammalian enzymes and loss of function of some members impairs human development^22^. Proline-rich inositol polyphosphate 5-phosphatase (PIPP, also known as INPP5J or PIB5PA) hydrolyzes PI(3,4,5)P_3_ to PI(3,4)P_2_ in breast tissue and melanocytes^23–25^. Unlike PTEN, few 5-phosphatases represent bone fide tumor suppressors^23,25,26^. However, the gene encoding PIPP (*INPP5J*), located on chromosome 22q12, is associated with allelic loss in ~30% of breast tumors^27,28^ and is one of the top 10 genes that predict breast cancer outcomes^29^. We reported that *Pipp* knockout mice exhibit normal mammary gland development and do not develop de novo mammary tumors up to 2 years of age^23^. However, *Pipp* ablation in the MMTV-*PyMT* oncogene-driven murine mammary cancer model enhances AKT activation and tumor growth, but paradoxically reduces lung metastasis via AKT1 activation^23^.

Individually, the PI-phosphatases PTEN and PIPP suppress breast cancer progression. However, the functional interplay between these enzymes, which hydrolyze the same substrate PI(3,4,5)P_3_ to different phosphoinositide species, has not been explored in vivo in breast cancer. Here, we demonstrate that PIPP and PTEN play non-redundant roles in suppressing mouse mammary epithelial cell transformation and disease progression in a subset of human breast cancers.

## Results

### *Pipp* ablation promotes end-stage lymphadenopathy in *Pten*^+/−^ mice

To explore the functional interplay between *PIPP* and *PTEN* in vivo, we generated mice with compound global deletions on a C57BL/6 background^23,30^. As homozygous *Pten* knockout mice are embryonically lethal^31^, we used heterozygous knockout mice (*Pten*^+/−^) that are viable but develop de novo tumors in a number of tissues^30^. Approximately 50% of female *Pten*^+/−^ mice develop mammary tumors which exhibit heightened AKT phosphorylation, between 10–12 months of age^32^. In addition, ~100% of female *Pten*^+/−^ mice show extensive lymphadenopathy by 20 weeks, due to impaired Fas-mediated apoptosis of T and B cells, leading to ethical endpoint at ~12 months^33^. Here, we crossed *Pipp*^−/−^ mice^23^ with *Pten*^+/−^ mice^30^ to generate compound *Pipp*^−/−^*;Pten*^+/−^ mice (Supplementary Fig. S1A). *Pipp*^−/−^*;Pten*^+/−^ mice were born at the expected Mendelian frequency and were indistinguishable from littermate controls (i.e., *Pten*^+/−^ or *Pipp*^−/−^ mice) at weaning. No significant differences in body weight were observed between genotypes at 5–7 months of age (Supplementary Fig. S1B, C). At ~5 months (150 days), no differences in relative organ weights between *Pipp*^−/−^ and wild-type mice (Supplementary Fig. S1D) were observed, consistent with the lack of an overt phenotype in *Pipp*^−/−^ mice^23^. By contrast, female *Pten*^+/−^ mice exhibited increased brain and liver weights relative to wild-type and *Pipp*^−/−^ mice and elevated spleen weight compared to *Pipp*^−/−^ mice (Supplementary Fig. S1D). *Pipp*^−/−^*;Pten*^+/−^ mice displayed increased liver and spleen weights compared to wild-type and *Pipp*^−/−^ mice and a small but significant decrease in relative heart weights compared to *Pipp*^−/−^ mice (Supplementary Fig. S1D). No differences were observed in relative organ weights between the *Pten*^+/−^ and *Pipp*^−/−^*;Pten*^+/−^ mice, except for the lungs at 210 days (Supplementary Fig. S1D, E).

Notably, *Pten*^+/−^ and *Pipp*^−/−^*;Pten*^+/−^female mice developed lymphadenopathy, predominantly in the submandibular, axillary and inguinal lymph nodes by 150 days, with no significant difference in the frequency between the two groups (Supplementary Fig. S1F). By 210 days, 100% of both *Pten*^+/−^ and *Pipp*^−/−^*;Pten*^+/−^ mice showed extensive lymphadenopathy. However, the development of end-stage lymphadenopathy (1 cm^3^ total burden) occurred significantly earlier in the female *Pipp*^−/−^*;Pten*^+/−^ mice (median survival 211 days) compared to *Pten*^+/−^ mice (246 days) (Supplementary Fig. S1G). No lymphadenopathy was detected in wild-type or *Pipp*^−/−^ mice (Supplementary Fig. S1F). These data suggest that loss of *Pipp* exacerbates *Pten*-induced lymphoproliferation and reduces overall survival.

### *Pipp* ablation in *Pten*^+/−^ mice enhances mammary gland hyperplasia, ductal multi-layering and cell proliferation

**Fig. 1:**
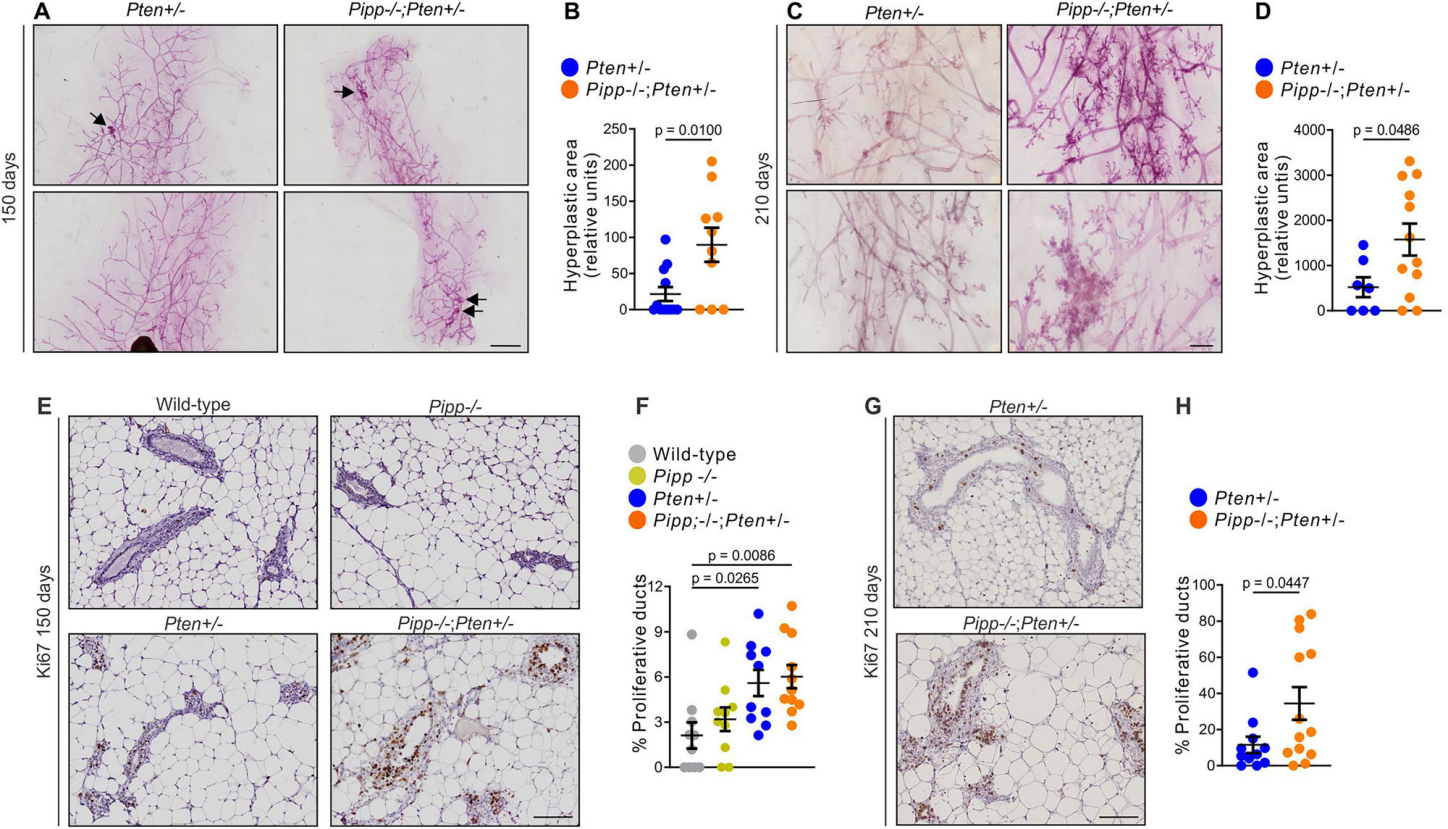
*Pipp* ablation promotes mammary gland hyperplasia in *Pten*^*+/−*^ mice. **A**–**D** Mammary gland whole mounts from *Pten*^*+/−*^ and *Pipp*^*−/−*^*;Pten*^*+/−*^ mice at 150 (**A**) and 210 (**C**) days of age were stained with carmine alum to detect hyperplastic foci (arrows). The total area of hyperplastic foci in the mammary glands was measured using Image J. Data represent the mean area of hyperplasia ± SEM from 150 d (**B**) (*Pten*^*+/−*^ n = 12 and *Pipp*^*−/−*^*;Pten*^*+/−*^ n = 10) and 210 d (**D**) (*Pten*^*+/−*^ n = 7 and *Pipp*^*−/−*^*;Pten*^*+/−*^ n = 12) mice. **E**–**H** Ki67 immunostaining of mammary gland sections from 150 d (**E**) wild-type, *Pipp*^*−/−*^, *Pten*^*+/−*^ and *Pipp*^*−/−*^*;Pten*^*+/−*^ mice and 210 (**G**) day old *Pten*^*+/−*^ and *Pipp*^*−/−*^*;Pten*^*+/−*^ mice. Data represent the mean percentage of proliferative ducts ± SEM, defined as ducts with >50% Ki67-positive cells from 150 d (**F**) (wild-type n = 10, *Pipp*^*−/−*^ n = 10, *Pten*^*+/−*^ n = 10 and *Pipp*^*−/−*^*;Pten*^*+/−*^ n = 11) and 210 d (**H**) (*Pten*^*+/−*^ n = 11 and *Pipp*^*−/−*^*;Pten*^*+/−*^ n = 13) mice. Scale bars, 2 mm (**A**), 500 μm (**C**), 100 μm (**E**, **G**).

Approximately 29% of *Pten*^+/−^ mice exhibit mammary gland hyperplasia by 12 months, and 15.5% develop adenocarcinomas^34^. *Pipp* ablation accelerates mammary gland hyperplasia in MMTV-*PyMT* mice^23^, therefore we investigated if co-loss of *Pten* accelerated this change. A significant increase in hyperplasia was detected at 150 (*Pten*^*+/−*^ 42% versus *Pipp*^*−/−*^*;Pten*^*+/−*^ 70%) and 210 (*Pten*^*+/−*^ 57% versus *Pipp*^*−/−*^*;Pten*^*+/−*^ 83%) days (Fig. 1A–D). Also, *Pten*^+/−^ and *Pipp*^−/−^*;Pten*^+/−^ murine mammary ducts exhibited increased cell proliferation compared to wild-type at 150 days of age (Fig. 1E, F) Furthermore, cell proliferation was significantly increased in *Pipp*^−/−^*;Pten*^+/−^ relative to *Pten*^+/−^ mammary glands at 210 days of age (Fig. 1G, H). No mammary tumors were detected in either *Pten*^+/−^ or *Pipp*^−/−^*;Pten*^+/−^ mice up to 210 days, and further ageing of the mice was not possible, as the ethical endpoint for lymphadenopathy was reached by ~7 months of age.

**Fig. 2:**
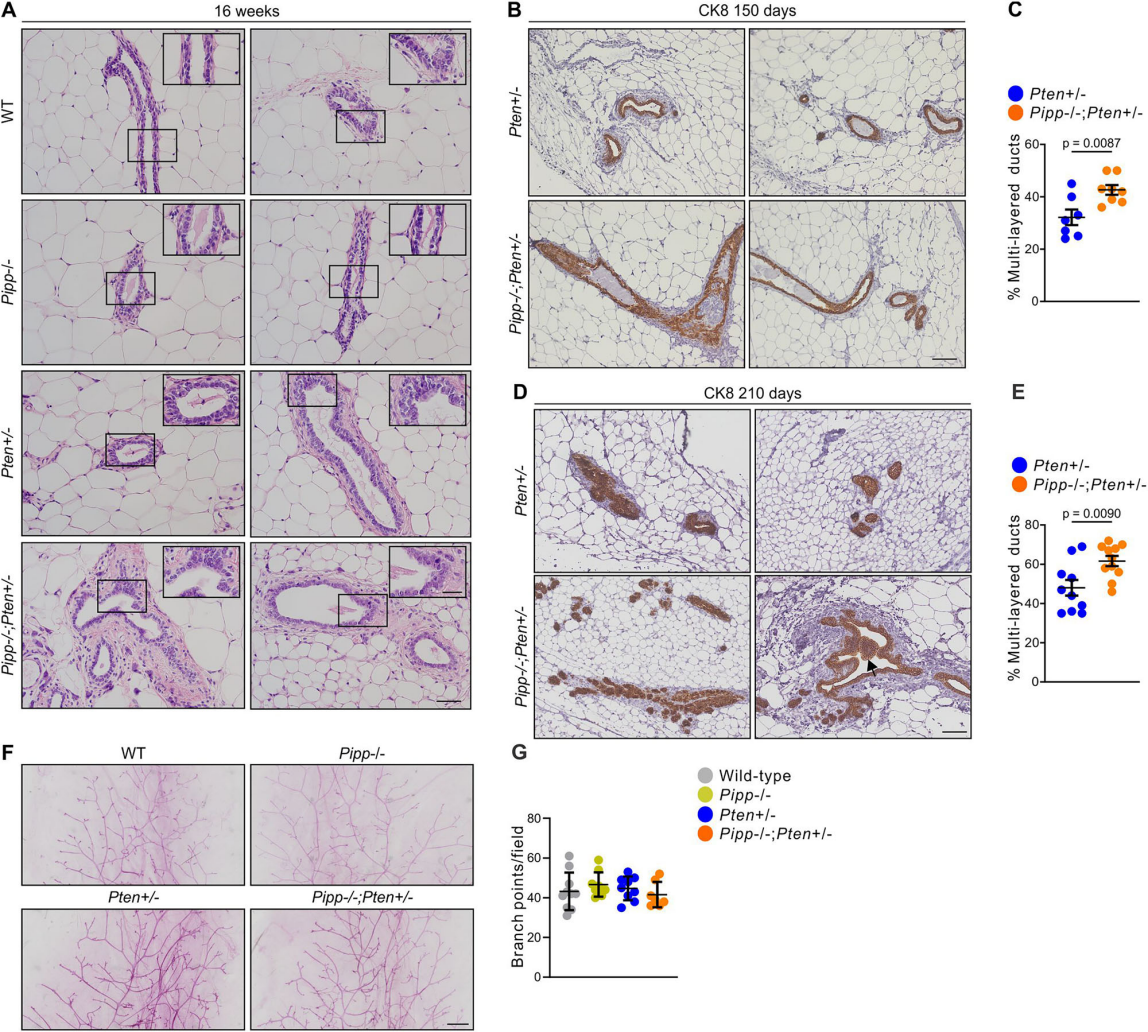
*Pipp* ablation promotes mammary ductal multi-layering in *Pten*^*+/−*^ mice. **A** FFPE sections of mammary glands from 16 w-old wild-type, *Pipp*^*−/−*^, *Pten*^*+/−*^ and *Pipp*^*−/−*^*;Pten*^*+/−*^ mice were stained with H&E. Higher magnification images of the boxed regions are shown. **B**–**E** FFPE sections of mammary glands from 150 d (**B**) and 210 d (**D**) *Pten*^*+/−*^ and *Pipp*^*−/−*^*;Pten*^*+/−*^ mice were immunostained with a cytokeratin 8 antibody. Arrow in (**D**) indicates intraductal luminal epithelial cells in the luminal space. Data represent mean percentage of multilayered ducts ± SEM from 150 d (**C**) (*Pten*^*+/−*^ n = 7 and *Pipp*^*−/−*^*;Pten*^*+/−*^ n = 8) and 210 d (**E**) (*Pten*^*+/−*^ n = 10 and *Pipp*^*−/−*^*;Pten*^*+/−*^ n = 12) mice. **F**, **G** Mammary gland whole mounts from wild-type, *Pipp*^−/−^, *Pten*^*+/−*^ and *Pipp*^*−/−*^*;Pten*^*+/−*^ mice at 150 days of age were stained with Carmine alum (**F**). The data represent the mean number of branch points ± SEM from 150 d (wild-type n = 10, *Pipp*^−/−^ n = 9, *Pten*^+/−^ n = 9 and *Pipp*^−/−^*;Pten*^+/−^ n = 7) (**G**) mice. Scale bars, 50 μm (**A**, 25 μm in higher magnification images), 100 μm (**B**, **D**), 500 μm (**F**).

Mouse mammary ducts are composed of a hollow lumen surrounded by a layer of luminal epithelial cells encased by a single layer of basal myoepithelial cells. Conditional murine homozygous knockout of *Pten* in mammary epithelial cells leads to precocious alveolar development, accelerated ductal extension, increased branching and development of mammary tumors from 2 months of age^35^. *Pten* ablation in stromal fibroblasts also accelerates mammary tumor initiation and progression in MMTV-*neu* oncogene-driven mice, associated with extracellular matrix remodeling^36^. *Pipp*^−/−^ mammary glands showed normal ductal morphology at 150 days of age (Fig. 2A) as reported^23^. By contrast, some mammary ducts from 150-day-old *Pten*^+/−^ and *Pipp*^−/−^*;Pten*^+/−^ mice exhibited epithelial cell multi-layering (Fig. 2A) consistent with enhanced cell proliferation. Multi-layering of CK8-positive luminal epithelial cells was detected in *Pten*^+/−^ mammary ducts and was further increased by *Pipp* ablation at 150 (Fig. 2B-C) and 210 days (Fig. 2D-E). Additionally, intraductal luminal epithelial cells were observed in the luminal space in *Pipp*^−/−^*;Pten*^+/−^ mammary ducts (Fig. 2D), which may represent an early step in mammary tumor development^37^. Conditional, homozygous *Pten* ablation in mammary epithelial cells results in precocious lobulo-alveolar development with excessive ductal branching^35^. Here, no significant differences in mammary ductal branching were observed between 150 d old *Pipp*^*−/−*^ and wild-type mice (Fig. 2F, G) as reported^23^. Heterozygous *Pten* ablation also had no effect on ductal branching, either alone or in combination with *Pipp* knockout (Fig. 2F, G).

**Fig. 3:**
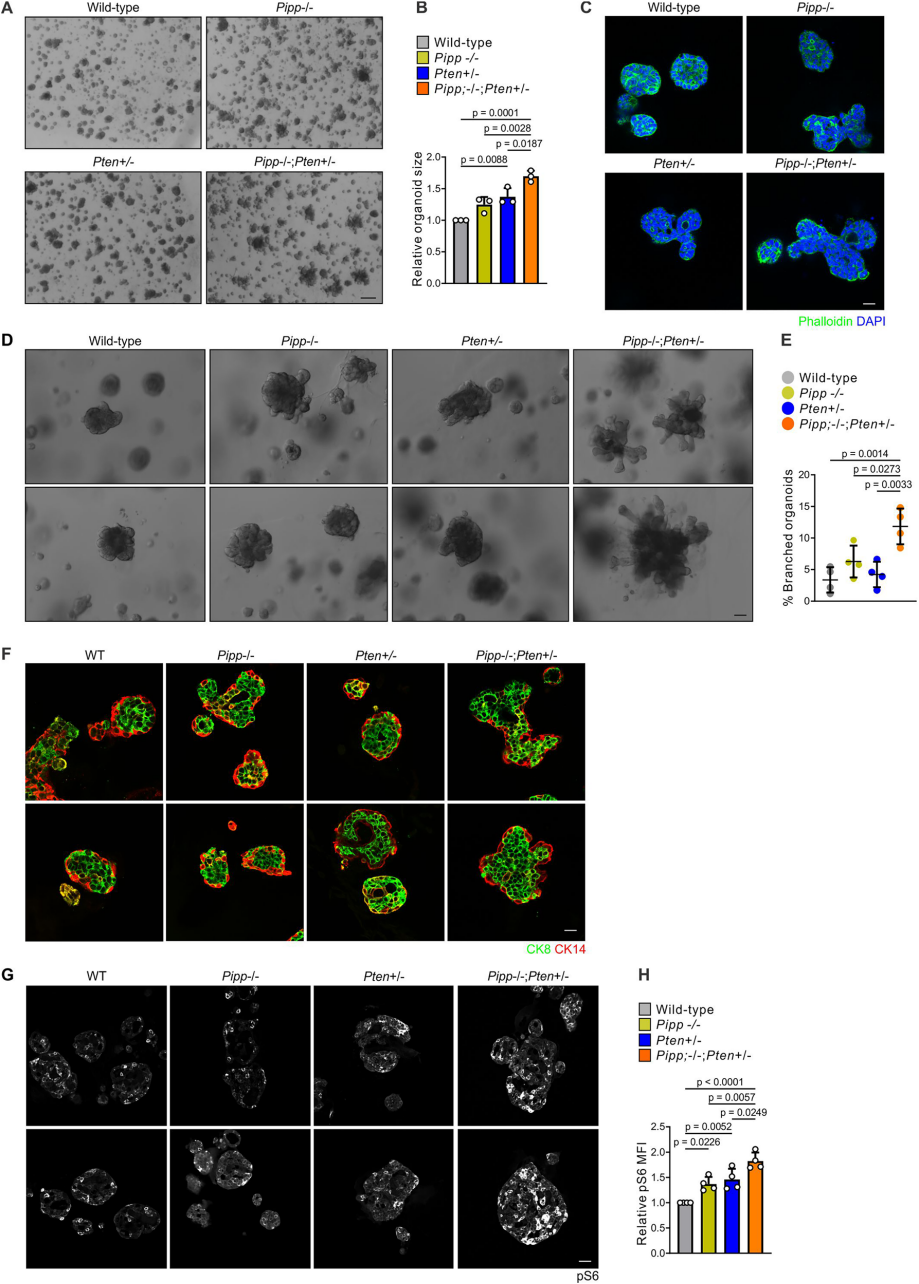
*Pipp* ablation increases the size of *Pten*^*+/−*^ mammary organoids. **A**, **B** Mammary organoids derived from wild-type, *Pipp*^*−/−*^, *Pten*^*+/−*^ and *Pipp*^*−/−*^*;Pten*^*+/−*^ mice were cultured in growth factor reduced Matrigel for 14 d and imaged by brightfield microscopy (**A**). Data represent the mean organoid size ± SEM relative to wild-type organoids which were arbitrarily assigned a value of 1 (**B**) (n = 3 independent experiments, >87 organoids/genotype/experiment). **C** FFPE sections of mammary organoids derived from wild-type, *Pipp*^*−/−*^, *Pten*^*+/−*^ and *Pipp*^*−/−*^*;Pten*^*+/−*^ mice cultured in Matrigel were stained with Alexa-Fluor 488 phalloidin and DAPI. **D**, **E** Mammary organoids derived from wild-type, *Pipp*^*−/−*^, *Pten*^*+/−*^ and *Pipp*^*−/−*^*;Pten*^*+/−*^ mice were cultured in growth factor-reduced Matrigel for 14 d and imaged by brightfield microscopy (**D**). Data represent the mean percentage of branched organoids ± SEM (**E**) (n = 4 independent experiments, 138-623 organoids/genotype/experiment). **F** FFPE sections of mammary organoids derived from wild-type, *Pipp*^*−/−*^, *Pten*^*+/−*^ and *Pipp*^*−/−*^*;Pten*^*+/−*^ mice cultured in Matrigel were immunostained with cytokeratin 8 and cytokeratin 14 antibodies. **G**, **H** FFPE sections of mammary organoids from wild-type, *Pipp*^*−/−*^, *Pten*^*+/−*^ and *Pipp*^*−/−*^*;Pten*^*+/−*^ mice were immunostained with a pS6 antibody (**G**). Data represent mean pS6 fluorescence intensity ± SEM (**H**) (n = 4 independent experiments, >138 organoids/genotype/experiment). Scale bars, 500 μm (**A**), 100 μm (**C**, **D**, **F**, **G**).

To further characterize the effects of *Pipp* and *Pten* loss, organoid cultures were established from murine mammary epithelial cells^38^. *Pipp*^−/−^*;Pten*^+/−^ mammary organoids were significantly larger than *Pipp*^−/−^ or *Pten*^+/−^ organoids (Fig. 3A, B) consistent with increased mammary epithelial cell proliferation. Wild-type, *Pipp*^−/−^ or *Pten*^+/−^ organoids showed rounded morphology with a limited number of lobules (Fig. 3C, D). By contrast, *Pipp*^−/−^*;Pten*^+/−^ mammary organoids exhibited a multi-lobular phenotype with a significant increase in the number of branched organoids compared to wild-type, *Pipp*^−/−^ or *Pten*^+/−^ (Fig. 3C–E), reminiscent of the large misshapen acini observed in MCF-10A cells expressing mutant *PIK3CA* or constitutively active AKT1^39,40^. There was no difference in the localization of CK8 and CK14 cells in organoids isolated from any genotype (Fig. 3F).

Dysregulation of the PI3K/AKT/p70S6K pathway is one of the most common events in breast cancer^41^. Staining for AKT phosphorylation itself was not effective in organoids due to the low reactivity of phospho-AKT antibodies in this experimental model. However, *Pipp*^−/−^ and *Pten*^+/−^ mammary epithelial organoids exhibited enhanced ribosomal protein S6 phosphorylation (pS6 staining intensity) relative to wild-type (Fig. 3G, H), suggesting elevated mTORC1 activity downstream of AKT. Furthermore, S6 phosphorylation was significantly increased in *Pipp*^−/−^*;Pten*^+/−^ mammary organoids compared to wild-type or *Pipp*^−/−^ or *Pten*^+/−^ alone (Fig. 3G, H) indicating that these PI-phosphatases function in a non-redundant manner to suppress mTORC1 signaling.

### *Pipp* ablation increases mammary gland fibrosis in *Pten*^+/−^ mice

**Fig. 4:**
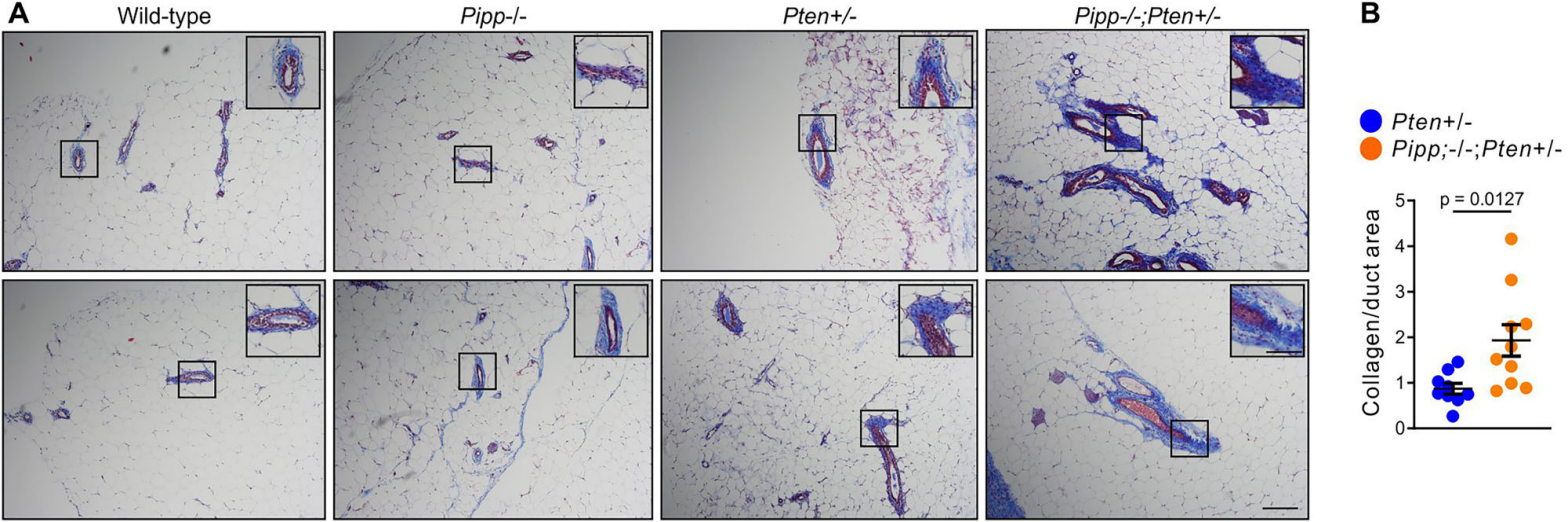
*Pipp* ablation enhances collagen deposition in *Pten*^*+/−*^ mammary ducts. **A** FFPE sections of mammary glands from 150 d-old wild-type, *Pipp*^*−/−*^, *Pten*^*+/−*^ and *Pipp*^*−/−*^*;Pten*^*+/−*^ mice were stained with Masson’s trichrome. Higher magnification images of the boxed regions are shown. **B** Data represent mean area of collagen staining/duct area ± SEM (*Pten*^*+/−*^ n = 9 and *Pipp*^*−/−*^*;Pten*^*+/−*^ n = 10 mice, 3–16 ducts/ section). Scale bar 100 μm (50 μm in higher magnification images).

The mechanical stiffness of the stromal extracellular matrix (ECM) is a key modulator of cell fate^42,43^. Increased matrix stiffness promotes mammary epithelial cell transformation and enhances tumor cell invasion and dissemination^44–46^. For example, *Pten* ablation in mammary stromal fibroblasts increases collagen I deposition within the mammary gland and promotes tumorigenesis in the MMTV-*neu* murine breast cancer model^36^. Histological (H&E) analysis of *Pten*^+/−^ and *Pipp*^−/−^*;Pten*^*+/−*^ 150-day-old mammary glands revealed that ducts were surrounded by areas resembling collagen deposition (Fig. 2A). Masson’s trichrome staining revealed a thin layer of collagen surrounding ducts in wild type mammary glands, which was increased in *Pten*^+/−^ ducts and further increased in *Pipp*^−/−^*;Pten*^+/−^ mammary ducts (Fig. 4A, B). These findings suggest that *Pipp* ablation enhances ductal cell proliferation in *Pten*^*+/−*^ mammary glands via cell autonomous and non-autonomous (i.e., matrix-mediated) mechanisms.

### Knockdown of both *PIPP* and *PTEN* enhances cell proliferation

We next established cellular models to evaluate PIPP/PTEN co-operation in mutant *PIK3CA-*dependent and independent contexts. Human breast cancer cell lines were screened for PI-phosphatase expression via droplet digital PCR and immunoblotting, which revealed the highest co-expression of *PIPP* and *PTEN* in T47D, ER+ breast cancer cells, with lower levels in non-transformed MCF-10A, ER + MCF-7, HER2 + SKBR3 and triple negative MDA-MB-231, Hs578T and SUM185PE cells (Supplementary Fig. S2). PTEN protein expression was not observed in breast cancer cell lines harboring a *PTEN* mutation (ZR-75-1, SUM149PT, BT549) (Supplementary Fig. S2B, C). We also investigated whether PIPP or PTEN expression or *PIK3CA* mutation correlated with AKT activation in human breast cancer cell lines following serum stimulation. High basal AKT activation was observed in *PIK3CA*^*H1047R*^ mutant T47D cells^47^ which was further increased upon serum stimulation (Supplementary Fig. S2B). By contrast, minimal AKT activation was observed in MCF-7 ER+ or SUM185PE triple negative cells harboring a *PIK3CA* mutation (Supplementary Fig. S2B, C). SUM185PE and T47D cells both harbor a *PIK3CA*^*H1047R*^ mutation. The differences observed in serum-stimulated AKT activation in these cell lines may reflect distinct cell line attributes such as hormone receptor expression in T47D cells or the presence of additional oncogenic mutations, including *FGFR3* and *BC2L1* amplification in SUM185PE cells^48^. Neither PIPP nor PTEN protein expression showed correlation with AKT signaling in ER+ or HER2+ cell lines (Supplementary Fig. S2B). However, high basal AKT activation was observed in *PTEN* mutant triple-negative breast cancer cell lines, which lacked PTEN protein expression (Supplementary Fig. S2C).

T47D (*PIK3CA*^H1047R^ mutation^47^) and Hs578T (*PIK3CA* wild-type) cells expressed both PIPP and PTEN and exhibited AKT activation following growth factor stimulation and therefore were selected for further studies. PIPP and PTEN were also expressed in MDA-MB-231 (*PIK3CA* wild-type) cells. Although minimal phosphorylated AKT was observed in MDA-MB-231 cells in response to serum stimulation, the downstream effector S6 was phosphorylated on residues Ser240/244 (Supplementary Fig. S2C), suggesting AKT activation in these cells. As we and others have reported single knockdown of *PIPP* or *PTEN* enhanced AKT activation in MDA-MB-231 cells in response to EGF stimulation or under basal conditions, respectively^23,49^, this cell line was also utilized in further studies.

**Fig. 5:**
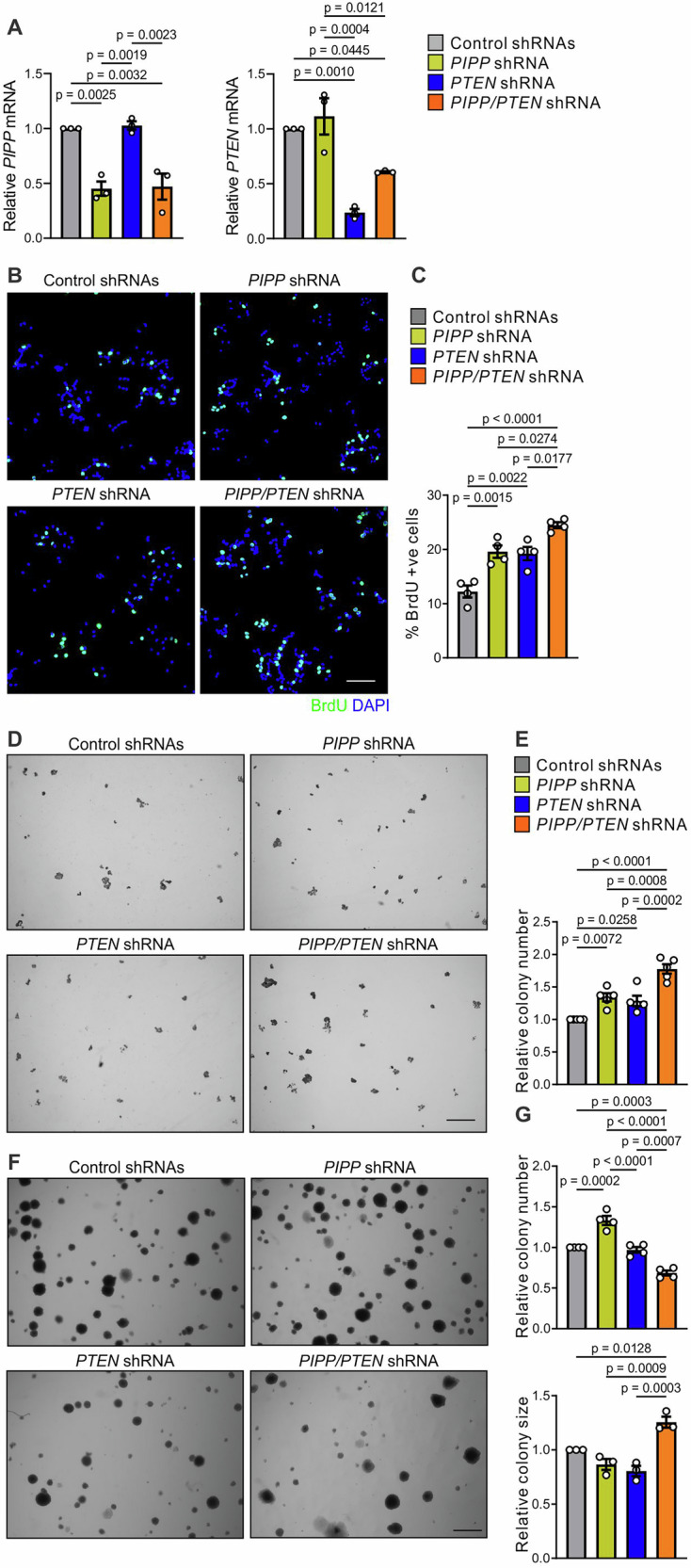
Co-shRNA knockdown of *PIPP* and *PTEN* enhances T47D breast cancer cell proliferation. **A** T47D cells were transduced with lentiviral particles encoding non-target control, *PIPP, PTEN* or *PIPP/PTEN* shRNA. RNA was extracted and subjected to two-step quantitative real-time PCR using primers for *PIPP* or *PTEN*. Expression was normalized to *GAPDH*. Expression was quantified from 3 independent experiments using the ΔΔCt method. Data represent mean transcript levels ± SEM, relative to T47D cells expressing control shRNAs which were arbitrarily assigned a value of 1. Significance was assessed via one-way ANOVA with Tukey’s Correction. **B**, **C** T47D cells expressing control, *PIPP*, *PTEN* or *PIPP/PTEN* shRNA were serum-starved for 24 h, incubated with BrdU for 45 min then fixed and stained with BrdU antibodies and DAPI (**B**). Data represent mean percentage of BrdU-positive cells ± SEM (n = 4 independent experiments, >1000 cells/experiment) (**C**). **D**, **E** T47D cells stably transduced with control, *PIPP*, *PTEN* or *PIPP/PTEN* shRNA were seeded into 6-well dishes (800 cells/cell line), cultured for 1 week then fixed, stained with DiffQuick and imaged via light microscopy (**D**). Data represent the number of colonies ± SEM relative to control shRNA-expressing T47D cells which were arbitrarily assigned a value of 1 (n = 4 independent experiments) (**E**). **F**, **G** T47D cells stably transduced with control, *PIPP*, *PTEN* or *PIPP/PTEN* shRNA were suspended in 0.3% agar and cultured for 4 weeks (**F**). Data represent the relative number of colonies (n = 4 independent experiments in triplicate) and relative colony size (n = 3 independent experiments in triplicate) (**G**) (n > 400 colonies/experiment) ± SEM. Scale bars, 100 μm (**B**), 2 mm (**D**, **F**).

To model loss of expression of *PTEN* and/or *PIPP* in vitro, short hairpin RNAs (shRNA) were utilized to stably knock down endogenous *PIPP* and/or *PTEN* in T47D, MDA-MB-231 and Hs578T cell,s resulting in 34–70% and 40–75% knockdown respectively, as assessed by quantitative real-time PCR (Fig. 5A and Supplementary Fig. S3).

Previous studies have reported that the individual knockdown of *PIPP* or *PTEN* increases AKT signaling and cell proliferation^23,50–52^. BrdU incorporation under serum-starved conditions was increased in both T47D and MDA-MB-231 cells with *PIPP* or *PTEN* knockdown alone, relative to non-target controls and was further increased with co-knockdown of both PI-phosphatases (Fig. 5B, C and Supplementary Fig. S4A, B). In control experiments, the increased proliferation of T47D-*PIPP/PTEN* shRNA cells was rescued by transient overexpression of exogenous HA-PIPP (Supplementary Fig. S4C). Clonogenic assays under anchorage-dependent conditions revealed increased colony numbers in T47D, MDA-MB-231 and Hs578T cells with knockdown of either *PIPP* or *PTEN* alone relative to non-target control cells, with a further increase with double *PIPP/PTEN* shRNA knockdown (Fig. 5D, E and Supplementary Fig. S4D, E, Supplementary Fig. S5A, B). However, colony number was not significantly increased in *PIPP/PTEN* shRNA co-knockdown MDA-MB-231 cells relative to *PTEN* knockdown alone (Supplementary Fig. S4D, E).

The ability of cells to grow in the absence of attachment is a feature of cell transformation. We reported *PIPP* shRNA knockdown in MDA-MB-231 or Hs578T triple-negative breast cancer cells enhanced anchorage-independent growth^23^. Here, T47D-*PIPP* shRNA cells exhibited a significant increase in the number of anchorage-independent colonies (Fig. 5F, G), although colony size was not increased relative to non-target shRNA control cells. By contrast, knockdown of *PTEN* in T47D cells had no effect on growth in soft agar (Fig. 5F, G), a result confirmed using two additional independent *PTEN* shRNAs (Supplementary Fig. S4F, G). Interestingly, co-knockdown of *PIPP/PTEN* in T47D cells resulted in fewer colonies relative to non-target control or single PI-phosphatase shRNA (Fig. 5F, G), reminiscent of the effects of *PIPP/PTEN* co-knockdown in melanoma cell lines^24^, which was attributed to activation of cell senescence. However, the colonies that did form were significantly larger than single PI-phosphatase knockdown or non-target controls (Fig. 5F, G), consistent with the enhanced cell proliferation.

*PIPP*-depletion impairs MDA-MB-231 breast cancer cell migration via AKT1^23^. In contrast, *PTEN* knockdown increases MCF-7 and MDA-MB-231 cell migration and invasion^49,53^. Here, MDA-MB-231-*PIPP* shRNA cells exhibited reduced migration towards a chemoattractant as reported^23^. *PTEN* knockdown alone, however, had no effect (Supplementary Fig. S4H-I), in contrast to the enhanced MDA-MB-231 cell invasion previously reported^49^. Here, only ~40% PTEN knockdown was achieved in these cells, mimicking the heterozygous state. MDA-MB-231-*PIPP/PTEN* knockdown cells exhibited impaired migration comparable to *PIPP* knockdown cells (Supplementary Fig. S4H, I), consistent with an interpretation that, in this context, PIPP but not PTEN regulates cell migration. T47D cells (expressing non-target control sRNA) showed limited migration towards a chemoattractant; therefore, the effects of *PIPP*/*PTEN* knockdown were not assessed in these cells.

### Co-knockdown of *PIPP* and *PTEN* increases AKT signaling

**Fig. 6:**
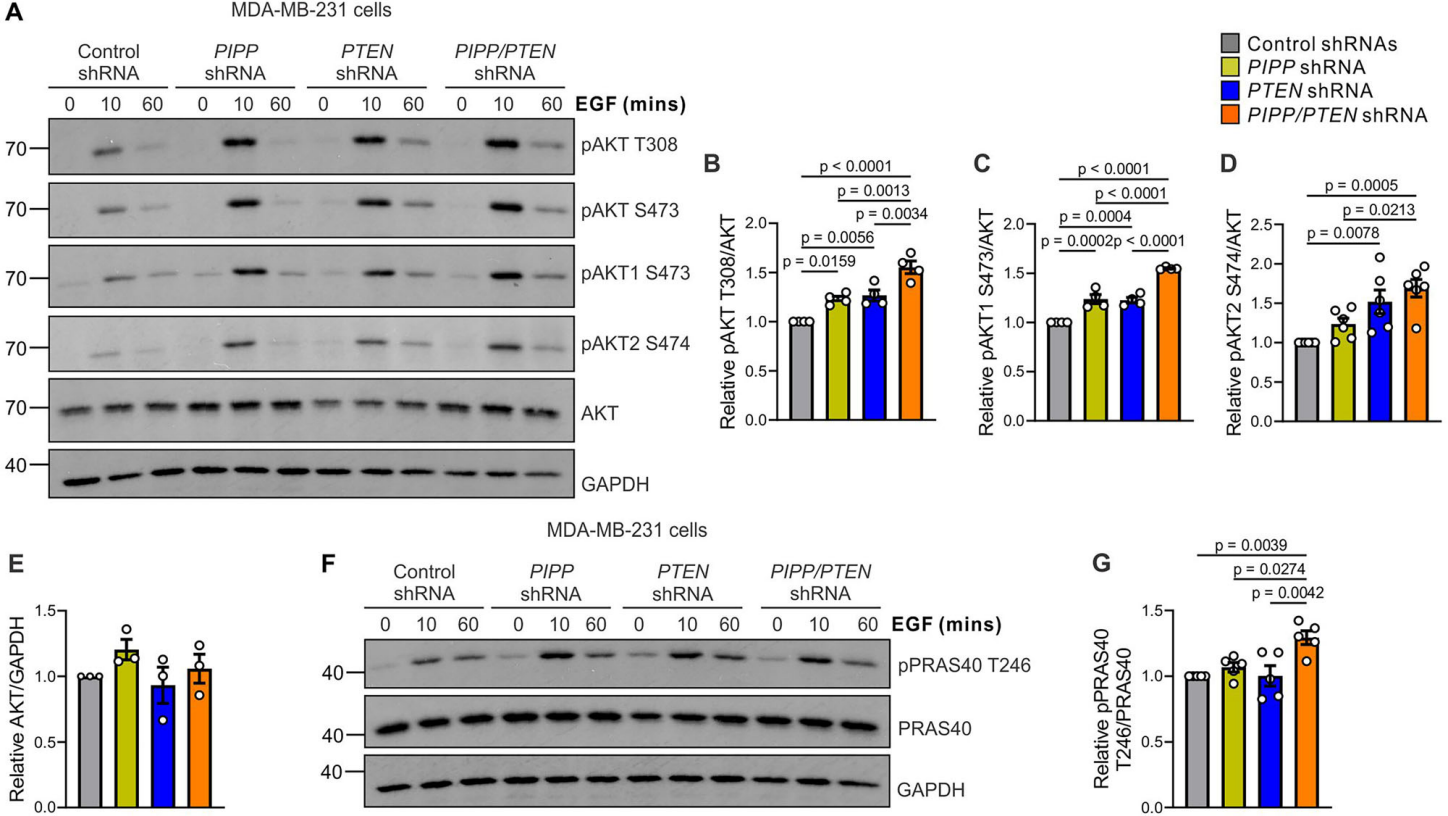
Co-shRNA knockdown of *PIPP* and *PTEN* enhances AKT signaling. **A**–**E** MDA-MB-231 *PIPP*, *PTEN, PIPP/PTEN* or control shRNA cells were serum starved overnight, stimulated with 100 ng/ml EGF for the indicated times then lysed and immunoblotted with pAKT Thr308, pAKT Ser473, pAKT1 Ser473, pAKT2 Ser474, AKT or GAPDH antibodies (**A**). Data represent mean pAKT Thr308 (n = 4) (**B**), pAKT1 Ser473 (n = 4) (**C**) or pAKT2 Ser474 (n = 6) (**D**) relative to AKT ± SEM or AKT relative to GAPDH (n = 3 independent experiments) (**E**) ± SEM. **F**, **G** MDA-MB-231 *PIPP*, *PTEN, PIPP/PTEN* or control shRNA cells were serum starved overnight, stimulated with 100 ng/ml EGF for the indicated times, then lysed and immunoblotted with pPRAS40 Thr246, PRAS40 or GAPDH antibodies (**F**). Data represent mean pPRAS40 Thr246 relative to PRAS40 (n = 5 independent experiments) (**G**) ± SEM.

PIPP and PTEN both dephosphorylate PI(3,4,5)P_3_ interconverting it to different species, PI(3,4)P_2_ and PI(4,5)P_2_, respectively, to suppress AKT1-3 activation. Here, we investigated whether co-knockdown of both PI-phosphatases further amplified AKT signaling above any single PI-phosphatase depletion following growth factor stimulation, using antibodies that detect Thr308 or Ser473-phosphorylated AKT1-3. Responses in MDA-MB-231 and Hs578T cells were examined. Phosphorylation of pan-AKT (Thr308 and Ser473) was enhanced in epidermal growth factor (EGF) stimulated MDA-MB-231 cells with knockdown of *PIPP* or *PTEN* relative to controls (Fig. 6A, B). In addition, *PIPP/PTEN* shRNA exhibited a further increase in EGF-stimulated pan-AKT signaling over either single PI-phosphatase knockdown or control in MDA-MB-231 cells (Fig. 6A, B). This analysis was repeated in Hs578T cells and revealed no significant difference in AKT activation in EGF-stimulated cells with knockdown of *PIPP* or *PTEN* relative to controls (Supplementary Fig. S5C, D). However, enhanced pan-AKT (Thr308 and Ser473) phosphorylation was observed in double *PIPP/PTEN* knockdown cells relative to single PI-phosphatase knockdown or controls (Supplementary Fig. S5C, D), indicating that loss of both PI-phosphatases enhances AKT activation in breast cancer cell lines.

Examination of only pan-AKT phosphorylation may obscure isoform-specific activation. Of the three AKT isoforms (AKT1, AKT2 and AKT3), AKT1 and AKT2 are the most prominently associated with human breast cancer initiation and progression^54^. For example, AKT1 mutation (E17K) occurs in 2-8% of human breast cancers and AKT2 is amplified in 2.8-4% of cases^6,54–59^. Here, the relative activation of AKT1 *versus* AKT2 was evaluated in MDA-MB-231 (Fig. 6A, C, D, E) and Hs578T cells (Supplementary Fig. S5E–G) using paralog-specific antibodies. In MDA-MB-231 cells, AKT1 phosphorylation was significantly enhanced with knockdown of either *PIPP* or *PTEN* relative to controls, whereas AKT2 activation was only significantly increased with *PTEN* depletion (Fig. 6A, C, D). However, double *PIPP/PTEN* knockdown enhanced AKT1 and AKT2 phosphorylation over single PI-phosphatase knockdown or controls in MDA-MB-231 cells (Fig. 6A, C, D). Neither AKT1 nor AKT2 phosphorylation was significantly altered with single PI-phosphatase knockdown in Hs578T cells relative to controls (Supplementary Fig. S5E–G) similar to results demonstrated with pan-AKT phosphorylation studies. Double knockdown of both PI-phosphatases in Hs578T cells increased both AKT1 and 2 isoform phosphorylation over controls and single knockdowns (Supplementary Fig. S5E–G) suggesting AKT suppression by these two PI-phosphatases is not isoform-specific. Increased phosphorylation of the AKT effector PRAS40 (Thr246) was observed in double *PIPP/PTEN* shRNA MDA-MB-231 cells relative to *PIPP* or *PTEN* knockdown, or controls (Fig. 6F, G). Collectively, for both cell lines loss of both PI-phosphatases further enhanced pan-AKT, AKT1 and AKT2 phosphorylation above that demonstrated with any single PI-phosphatase depletion.

### Reduced *PIPP* and *PTEN* mRNA expression occurs in a subset of human breast cancers, associated with reduced survival

**Fig. 7:**
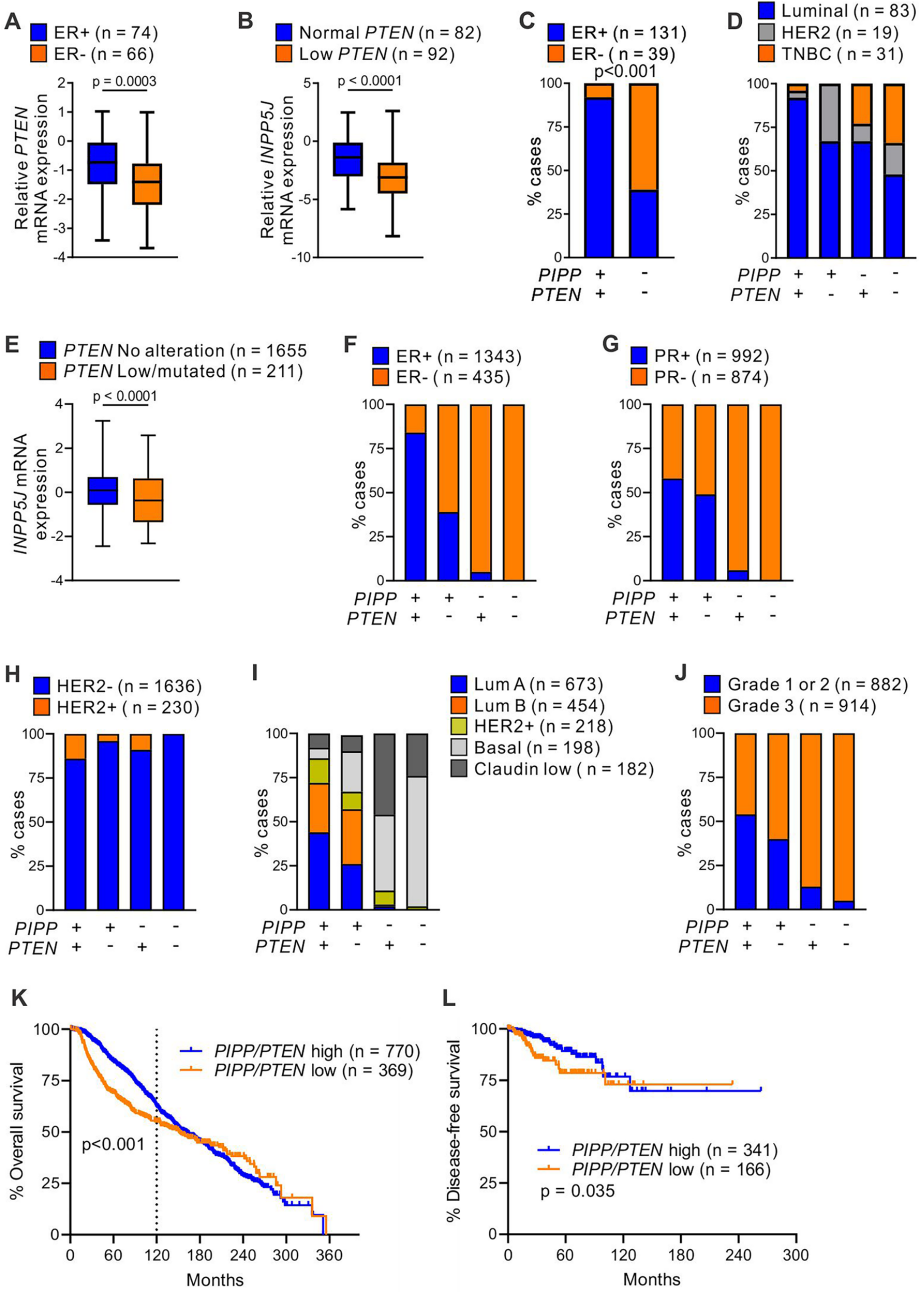
Low *PIPP/PTEN* mRNA expression correlates with reduced survival in breast cancer patients. **A** Normalized *PTEN* mRNA expression was determined by qPCR using TissueScan Breast Cancer Arrays I-IV with *PTEN* and β-actin primers. The data are displayed as box and whiskers on a log scale. The center line indicates the median; the box extends from the 25th to 75th percentiles and the whiskers extend from the minimum to maximum values. *PTEN* mRNA expression was correlated with ER (140 cases), p values were determined using an unpaired t test. **B** *PIPP* mRNA expression was correlated with normal versus low *PTEN* mRNA expression in TissueScan Breast Cancer Arrays I-IV (Normal *PTEN* expression n = 82; low *PTEN* mRNA (2-fold reduction relative to normal breast tissue) n = 92). The data are displayed as box and whiskers on a log scale. The center line indicates the median; the box extends from the 25th to 75th percentiles, and the whiskers extend from the minimum to maximum values. **C** Breast cancer cases in TissueScan Breast Cancer Arrays I-IV were scored for normal (*PIPP* + */PTEN* + ) versus low (*PIPP-/PTEN-*) *PIPP* and *PTEN* mRNA expression in ER+ and ER– tumors (170 cases). Significance was determined using a two-sided Fisher’s exact test (p < 0.001). **D** Breast cancer cases in TissueScan Breast Cancer Arrays I-IV were scored for normal versus low *PIPP* and *PTEN* mRNA expression relative to breast cancer subtype (133 cases). **E** *PIPP* mRNA expression was correlated with *PTEN* alterations in the METABRIC dataset. *PIPP* expression was correlated with unaltered *PTEN* versus altered *PTEN* (mutated and/or low expression). The data are displayed as box and whiskers. The center line indicates the median; the box extends from the 25th to 75th percentiles, and the whiskers extend from the minimum to maximum values. p-values were determined using an unpaired t-test. **F**–**J** Breast cancer cases in the METABRIC dataset were scored for reduced *PIPP* expression and/or *PTEN* expression (Z-score threshold of <1.5 relative to all breast cancers in the cohort) and/or *PTEN* mutation and correlated with ER (**F**), PR (**G**), HER2 (**H**), breast cancer subtype (**I**) or tumor grade (**J**). **K** Overall survival analysis in breast cancer patients using the METABRIC dataset. Samples were dichotomized for high *PIPP/PTEN* gene expression (>40th percentile) versus low *PIPP/PTEN* gene expression (<40th percentile). Statistical significance for 10-year survival was determined using a log-rank test. **L** Disease-free survival analysis in breast cancer patients using the TCGA Pan Cancer dataset. Samples were dichotomized for high *PIPP/PTEN* gene expression (>40th percentile) versus low *PIPP/PTEN* gene expression (<40th percentile). Statistical significance was determined using a log-rank test.

The relative expression of *PIPP versus PTEN* was examined in human breast cancer datasets. As reported, *PIPP* (*INPP5J*) mRNA expression was reduced in ER– relative to ER+ breast tumors based on analysis of 176 human cancers and 16 normal, adjacent breast tissues using Tissue Scan Breast Cancer cDNA array I-IV (OriGene)^23^. Moreover, *PIPP* expression was significantly decreased in triple-negative breast cancers relative to normal breast or luminal breast cancers^23^. Here, analysis of the same breast cancer cohort revealed lower *PTEN* mRNA expression in ER– relative to ER+ tumors (Fig. 7A). In addition, *PTEN* mRNA expression was lower in HER2+ and triple negative compared to luminal breast cancers and was significantly decreased in all breast cancer subtypes relative to normal breast tissue (Supplementary Fig. S6A).

*PIPP* mRNA expression was also assessed in cancers in the Tissue Scan Breast Cancer cDNA array I-IV stratified for normal *versus* low *PTEN* expression (defined as a 2-fold reduction in *PTEN* mRNA relative to normal breast tissue). Interestingly, low *PIPP* was significantly associated with low *PTEN* mRNA expression (Fig. 7B). Tumors in this cohort were also stratified for >2-fold reduction in *PIPP* and *PTEN* expression (*PIPP*–/*PTEN*–) relative to normal breast tissue. Approximately half of all breast cancers exhibited low expression of both *PIPP* and *PTEN* (83/174 tumors), with 21% (37/174) showing comparable expression of both PI-phosphatases relative to normal breast tissue (Supplementary Fig S6B). Tumors with *PIPP/PTEN* at “normal” levels were predominantly ER+ (92%). By contrast, only 39% of tumors with low *PIPP/PTEN* were ER+ (Fig. 7C). *PIPP* + */PTEN*+ and *PIPP*–*/PTEN*– tumors were also stratified according to tumor subtype and grade. Tumors with co-reduction of the two PI-phosphatases were predominantly of the luminal (48%) or triple negative (34%) subtypes, with 18% HER2+ breast cancers (Fig. 7D). The *PIPP/PTEN* reduced expression cohort was associated with grade 3 breast cancers (Supplementary Fig. S6C), suggesting that expression of the two PI-phosphatases is reduced in more undifferentiated tumors.

PTEN inactivation can occur by several mechanisms, including loss of expression and somatic mutations. Examination of the METABRIC (Fig. 7E) and TCGA (Supplementary Fig. S6D) datasets revealed *PIPP* expression was significantly lower in cancers with low *PTEN* and/or *PTEN* mutation. In these cohorts, tumors with reduced *PIPP* and *PTEN* expression (Z-score threshold of <1.5 relative to all breast cancers in the cohort) and/or *PTEN* mutation (designated *PIPP*–*/PTEN*–) were almost exclusively hormone receptor and HER2 negative (METABRIC: ER–100%, PR–100%, HER2–100%; TCGA: ER–92%, PR–96%, HER2–94%) (Fig. 7F–H, Supplementary Fig. S6E–G) and of the basal or claudin low subtype (METABRIC: 98%) (Fig. 7I). Similar to the Tissue Scan Breast Cancer cDNA array I-IV, *PIPP*–*/PTEN*– tumors in the METABRIC cohort were predominantly grade three breast cancers (Fig. 7J). Notably, low *PIPP/PTEN* predicted for reduced overall survival within 10 years of diagnosis (Fig. 7K) and decreased disease-free survival in all breast cancers (Fig. 7L). These data collectively suggest that a reduction in both *PTEN* and *PIPP* expression is observed in multiple breast cancer subtypes and predicts for reduced long-term survival.

## Discussion

Here, we used several experimental models of breast cancer, both dependent and independent of *PIK3CA* mutation, to demonstrate that loss of both the PI(3,4,5)P_3_ phosphatases, PTEN and PIPP, compared to single phosphatase loss, has an additive effect on PI3K/AKT signaling and cell proliferation. PI(3,4,5)P_3_ is hydrolyzed at either the 3’ or 5’ hydroxyl positions of the inositol ring by PTEN and PIPP, respectively. Loss of both PI-phosphatases increases PI3K/AKT/PRAS40 signaling, breast cancer cell proliferation and colony growth in *PIK3CA* wild-type (MDA-MB-231, Hs578T) and mutant (T47D) cell lines above that seen with loss of an individual PI-phosphatase. In addition, *Pipp* ablation in *Pten*^*+/−*^ mice promotes mammary epithelial cell proliferation associated with increased hyperplasia and ductal multi-layering, independent of *PIK3CA* mutation. These findings are consistent with the observation that PIPP and PTEN expression is reduced in approximately half of all primary human breast cancers, associated with reduced overall survival. Therefore, we propose PTEN and PIPP play non-redundant roles in regulating AKT/PRAS40 signaling in breast cancer cells, which may affect long-term outcomes following their loss of expression.

Studies in genetically engineered mice using a series of targeted *Pten* alleles that varied *Pten* levels in vivo revealed a tight correlation between *Pten* protein expression and function^60^. Furthermore, in murine prostate tissue, a progressive lowering of PTEN protein had an additive effect on AKT activation, prostate enlargement, proliferation of prostate cells and tumorigenesis^61^. We propose the functional effect of *PIPP* loss in the context of *PTEN* deficiency also has an additive effect on AKT signaling and thereby breast cancer cell proliferation. PIPP does not appear to be a significant tumor suppressor in its own right. For example, *Pipp*^*−/−*^ mice are viable and have a normal life span^23^, which does not phenocopy the early mortality and breast cancer incidence in *Pten*^*+/−*^ mice^34^. However, mammary gland hyperplasia was significantly increased in *Pipp*^*−/−*^*;Pten*^*+/−*^ mice consistent with the increased proliferation observed with loss of both PI-phosphatases in breast cancer cell lines. As *Pipp*^*−/−*^*;Pten*^*+/−*^ mice developed end-stage lymphadenopathy by ~7 months of age, examining mammary tumor growth was not feasible and ideally in future studies we will examine the effects of *Pipp* deletion in mammary-specific *Pten*^*−/−*^ mice. However, breast cancer organoid model studies reported here are also consistent with an interpretation of non-redundancy for PTEN and PIPP in the regulation of breast cancer cell proliferation. Furthermore, our human dataset analyses reveal that loss of *PIPP* in human breast cancer frequently co-occurs with loss of *PTEN*, with worse outcomes, supporting the threshold model of cancer progression. Therefore, collectively our findings from human breast cancer analysis, and cell and animal models of *Pipp* and *Pten* loss, predict that loss of *PIPP* expression is one of several cooperative steps towards tumor formation in breast cancer in the context of *PTEN* deficiency.

The AKT1 and AKT2 isoforms play dominant roles in breast cancer. AKT kinases are recruited to membranes and activated by PI(3,4,5)P_3_ and/or PI(3,4)P_2_. Recent studies have suggested AKT isoforms are differentially activated in an isoform- and site-specific manner. AKT1 is selectively activated by PI(3,4,5)P_3_ at the plasma membrane, whereas PI(3,4)P_2_ stimulates AKT2 at both the plasma membrane and early endosomes^62^. INPP4B, which degrades PI(3,4)P_2_ at the 4-position on the inositol ring, on early and late endosomes, selectively inhibits AKT2 activation on early endosomes in thyroid cancer cells^63^. AKT2 but not AKT1 drives thyroid tumorigenesis in *Inpp4B*^*Δ/Δ*^*;Pten*^*+/−*^ mice^63,64^. Here, we demonstrated *PIPP/PTEN* co-depletion further enhanced AKT1 and AKT2 activation in response to growth factor simulation of breast cancer cell lines over single knockdown of either PI-phosphatase. Organoids derived from *Pipp*^*−/−*^*;Pten*^*+/−*^ mammary epithelial cells exhibited an enlarged, multilobular phenotype. MCF-10A cells expressing mutant *PIK3CA* or constitutively active AKT1 also form large, misshapen acini reminiscent of the aberrant glandular structures in premalignant tumors^39,40^. Active AKT1 enhances proliferation during the early stages of MCF-10A acini morphogenesis^40^. Both AKT1 and AKT2 are required for *PTEN*-deficient prostate cancer spheroid formation but only AKT2 is essential for tumor spheroid maintenance^65^. The multi-acinar phenotype of *Pipp*^*−/−*^*;Pten*^*+/−*^ mammary organoids observed here is consistent with hyperactivated PI3K/AKT signaling driving enhanced cell proliferation, although further investigation is required to determine if this is restricted to early organoid development. Interestingly, we previously demonstrated that *PIPP* deletion suppresses cell migration and thereby lung metastasis via AKT1 hyperactivation^23^. Here, although we demonstrated that co-*PIPP* and *PTEN* loss increased AKT activation, cell migration was not affected over *PIPP* knockdown alone. As our human database analysis suggests loss of both PI-phosphatases leads to poor outcomes, it is therefore likely that this is independent of cell migration.

Although PIPP and PTEN both hydrolyze PI(3,4,5)P_3_, these reactions generate different products, PI(3,4)P_2_ and PI(4,5)P_2_, respectively. Complexity arises as PTEN also degrades PI(3,4)P_2_ and PIPP hydrolyzes PI(4,5)P_2_, with both reactions generating PI(4)P. Furthermore, PI(4,5)P_2_ can be hydrolyzed by phospholipase C to generate diacylglycerol and inositol 1,4,5-trisphosphate, which can also be hydrolyzed by PIPP to inositol 1,4-bisphosphate^66^. Co-depletion of these two PI-phosphatases will increase PI(3,4,5)P_3_ with effects on PI(3,4)P_2_, PI(4,5)P_2_, PI(4)P and inositol phosphates less clear. Mass spectrometry and PI-specific biosensors^67,68^ could be utilized to address how co-depletion of *PIPP* and *PTEN* affects the interplay between PI substrates and products. However, irrespective of this information, which of any one PI or inositol phosphate species facilitates the functional phenotypes observed with co-depletion of *PIPP* and *PTEN* will be complex, as changes to many PIs and inositol phosphates may contribute to the observed functional changes.

There is a growing appreciation of the role the tumor microenvironment plays in breast cancer initiation and progression. As the mouse models used here were global knockout mice, the effects observed in *Pipp*^*−/−*^*;Pten*^*+/−*^ mammary glands could be cell autonomous and/or stromal-dependent. Genetic inactivation of *Pten* specifically in mammary gland stromal fibroblasts accelerates neu oncogene-driven mammary epithelial tumor initiation and growth via an Ets2-regulated transcriptional program^36^. In addition, stroma-specific *Pten* knockout in mice (*Fsp-Cre;Pten*^*loxP/loxP*^) increases AKT and JNK signaling in both the stroma and adjacent epithelial cells, as well as enhancing Type I collagen deposition and innate immune cell infiltration^36^. Here, increased collagen deposition was observed in *Pipp*^*−/−*^*;Pten*^*+/−*^ *versus Pten*^*+/−*^ mammary glands. Collagen contributes to high mammographic density, a risk factor for breast cancer and collagen deposition in the mammary gland promotes breast cancer development and progression^45^. *Pten* knockout in mammary fibroblasts promotes collagen shuttling out of the cell and *Fsp-Cre;Pten*^*loxP/loxP*^ mice exhibit increased collagen fiber number and size within the mammary gland^69^. Interestingly, *PIPP*-deficient breast cancer cells exhibit decreased levels of the matrix metalloproteinase MMP2, which digests collagen type IV^23^, suggesting that PIPP may also regulate extracellular matrix remodeling. However, further investigation is required to delineate the effects of PIPP and its cooperativity with PTEN in mammary epithelial cells *versus* the stroma.

Pregnancy causes substantial changes in the mammary gland, including epithelial cell expansion and stromal remodeling and is associated with a transient increase in breast cancer risk^70,71^. When lactation ceases, the mammary gland returns to a pre-pregnancy state via involution, which utilizes tissue-remodeling programs reminiscent of wound healing, a pro-oncogenic process^71^. Conditional mammary epithelial cell-specific *Pten* ablation promotes lobulo-alveolar development and hyperplastic growth during pregnancy and impairs involution following weaning^35^. Furthermore, increased tumor incidence with decreased latency is observed in multiparous mice^35^. Although global *Pipp* ablation does not affect mammary gland remodeling during pregnancy or involution^23^, it would be of interest to examine the impact of ablation of both PI-phosphatases on pregnancy-associated breast cancer development in future studies.

It will be particularly interesting to dissect the role of PIPP in other cancers such as melanoma, given others have reported that PIPP has oncogenic potential in melanoma via enhanced AKT/mTOR signaling^24^. Further studies using tissue-specific deletion of *Pipp* and *Pten* may reveal the role these PI-phosphatases play in controlling tumor progression and metastasis in other cancer contexts. *PIPP* is frequently downregulated in melanoma via loss of DNA copy number or histone hypoacetylation, and approximately one-third of *PTEN*-null melanomas exhibit *PIPP* deficiency^24^. Furthermore, high levels of miR-508, a microRNA which targets multiple phosphatases including *PIPP*, *PTEN* and *INPP4A*, correlate with clinical stage and reduced 5-year disease-free and overall survival in esophageal squamous cell carcinoma patients^72^.

The most common driver of ER+ breast cancer is *PIK3CA*, the catalytic subunit of phosphoinositide 3-kinase (PI3K), mutated in 40% of ER+ breast cancers^4,5^. Despite immense efforts by pharma, PI3K inhibitors have limited clinical impact due to toxicity and insulin feedback loops. They are currently used only for relapsed ER+ breast cancer, but treatment inevitably fails^73^. However, PI3K signaling complexity is still emerging. As shown here, PI3K/AKT signaling is further enhanced by loss of PTEN and PIPP over and above that observed with loss of an individual PI-phosphatase in breast cancer cells, and future screening for these changes in breast cancer cohorts may contribute to improved patient selection for treatments.

## Materials and methods

### Materials

Antibodies were from: pAKT^Thr308^ (#2965), pAKT^Ser473^ (#4058), AKT (#4685), pAKT1^Ser473^ (#9018), pAKT2^Ser474^ (#8599), pPRAS40^Thr246^ (#2997), PRAS40 (#2691), pS6^Ser235/236^ (#4858), pS6^Ser240/244^ (#5364), HA (#3724), PI3K p110α (clone C73F8, #4249), PTEN (clone 138G6, #9559) Cell Signaling Technology (Boston, MA, all diluted 1/1000); Ki67 (RM-9106-SO, diluted 1/800), GAPDH (#AM4300, diluted 1/40,000), PIPP (#PA104005) ThermoFisher Scientific (Waltham, MA, diluted 1/1,000); CK8 (#2031-1, diluted 1/1000) Epitomics; CK14 (#ab7800, diluted 1/2000) Abcam (Cambridge, MA); ACTIN (#MA5-11869) Neomarkers (ThermoFisher Scientific, diluted 1/1000); HRP-conjugated secondary antibodies (Merck Millipore, Burlington, MA, diluted 1/10,000); fluorescently labeled secondary antibodies (diluted 1/800) and Alexa-Fluor 488 phalloidin (diluted 1/1000) Molecular Probes (ThermoFisher Scientific). DAPI was from Sigma-Aldrich (Merck Millipore, Burlington, MA, 1 μg/ml). Recombinant human insulin was from Sigma-Aldrich, and recombinant epidermal growth factor was from BD Biosciences.

### Generation of *Pipp*^*−/−*^*;Pten*^*+/−*^ mice

All procedures involving mice were approved by the Monash University Animal Ethics Committee, Monash University, Australia (Project Numbers MARP/2013/108, MARP/13267, MARP/28260). We have complied with all relevant ethical regulations for animal use.

*Pipp*^−/−^ mice^23^ and *Pten*^+/−^ mice^30^ (provided by Dr Antonella Papa, Monash University) on a C57Bl/6 background have been described previously. *Pipp*^*+/−*^*;Pten*^*+/−*^ mice were generated by crossing female *Pipp*^*−/−*^ mice with male *Pten*^*+/−*^ mice. To increase the efficiency of generating experimental wild-type, *Pipp*^−/−^, *Pten*^+/−^ and *Pipp*^*−/−*^*;Pten*^*+/−*^ mice, female *Pipp*^+/+^ and *Pipp*^−/−^ mice were crossed with male *Pipp*^+/+^*;Pten*^+/−^ and *Pipp*^−/−^*;Pten*^+/−^ mice, respectively. Twelve-week virgin female mice of all genotypes were used for establishing organoid cultures and 5–7-month-old virgin female mice were used in all other experimental studies. Mice were group housed where possible with a 12–12 h light-dark cycle, 22–24 °C and were fed a commercial diet ad lib.

The Institutional Animal Care and Use Committee allowed a maximum cumulative tumor/lymphadenopathy volume of 1000 mm^3^ per mouse. This limit was not exceeded in any of the experiments. Mice with lymphadenopathy were monitored at least three times per week. Mice were euthanized by CO_2_ inhalation followed by cervical dislocation. Sample sizes were established based upon the investigators’ prior experimental experience, and exact numbers of mice used for each experiment are detailed in the corresponding figure legends.

The genotype of mice was verified by PCR analysis of genomic DNA using the following primers: *Pipp* WT 5’: GCT AAT GGA CCT ACT TTG GAA CCC TG, *Pipp* KO 5’: GAT CAG CAC AGG CAG GGC TGT GAC, *Pipp* 3’: CAC ATT CCA TGT AAC CAC AGT GAT C; *Pten* Pgen-1: TGG GAA GAA CCT AGC TTG GAG G, *Pten* Pgen-3: ACT CTA CCA GCC CAA GGC CCG G, *Pten* 3193: CGA GAC TAG TGA GAC GTG CTA CTT CC.

### Randomization

Environmental conditions, including lighting, temperature, and humidity, were kept consistent for all animals, and the cages were randomly positioned within the rack. Genetically modified cells in each experiment were derived from the same pool of parent cells.

### Blinding

The analysts were blinded to mouse genotype during data assessment. There was no blinding in other experiments as the same investigator performed the experiment and analyzed the data.

### Inclusion and exclusion criteria

Genotype and sex were the only selection criteria used for assigning mice to experiments, and no other variables were considered. No data was excluded.

### Mammary fat pad Carmine alum staining

Inguinal mammary fat pads were removed from mice, spread onto Superfrost slides and fixed with Carnoy’s fixative (60% ethanol, 30% chloroform, 10% glacial acetic acid) overnight. Mammary fat pad whole mounts were stained with Carmine alum (StemCell Technologies, Vancouver, BC, Canada) according to the manufacturer’s instructions, cleared in xylene and coverslips mounted with Permount (ThermoFisher Scientific). Slides were imaged using an Olympus dotSlide microscope with a 2x objective. The total hyperplastic area in each mammary fat pad whole mount was measured using ImageJ. The total number of branch points was counted in a box of defined area next to the lymph node, distal to the nipple.

### Mammary gland histology

Following dissection, murine inguinal mammary glands were fixed in 10% formalin and embedded in paraffin. Mammary gland morphology and fibrosis (collagen deposition) were examined by staining 5 µm thick sections with hematoxylin & eosin (H&E) or Masson’s trichrome, respectively, using standard procedures. Mammary gland histology was performed by the Monash Histology Platform (Monash University, Australia). Sections were imaged using an Olympus Provis light microscope with a ×20, ×40 or ×100 oil immersion objective (Monash Micro Imaging, Monash University, Australia).

### Mammary gland immunohistochemistry

5 µm formalin fixed, paraffin embedded (FFPE) mammary gland sections were dewaxed in three changes of xylene then rehydrated in three changes of ethanol. Heat-induced antigen retrieval was performed in a pressure cooker for 10 min in Novocastra Epitope Retrieval solution pH 9 (Ki67, CK8) or 10 mM sodium citrate pH 6 (pS6) antigen retrieval buffer. Sections were blocked in 1% BSA/50 mM Tris/150 mM NaCl, pH 8 for 1 h, then incubated with primary antibodies diluted in blocking buffer at 4 °C overnight. Following 3 washes in 50 mM Tris/150 mM NaCl, pH 8, endogenous peroxidase activity was quenched with 0.3% hydrogen peroxide for 10 min prior to incubation with EnVision^+^ HRP-conjugated antibodies (Dako Agilent, Santa Clara, CA) for 1 h at room temperature. Immunoreactivity was detected by DAB staining (Dako Agilent, Santa Clara, CA). Sections were counterstained with hematoxylin, then dehydrated in three changes of ethanol followed by three changes of xylene. Coverslips were mounted using DPX mounting reagent. Sections were imaged using an Olympus dotSlide microscope (Monash Micro Imaging, Monash University, Australia).

### Mammary organoid culture

Fourth mammary fat pads from ~12-week-old virgin mice were minced with a scalpel blade, then incubated in 10 ml digestion solution (DMEM/F12 media supplemented with 300 U/ml collagenase III, 0.25% (v/v) trypsin, 5% (v/v) FCS, 50 μg/ml gentamycin) at 37 °C for 30 min in an orbital shaker. Fragments were enriched by centrifugation at 1250 x *g* for 10 min and the top layer (including the fat layer) was transferred to a fresh tube containing 5 ml DMEM/F12 + 5% (v/v) FCS. 5 ml DMEM/F12 + 5% (v/v) FCS was also added to the bottom layer. Fragments were resuspended by vigorous pipetting then centrifuged at 1250 x *g* for 10 min. Supernatants were aspirated and fragments from both layers were resuspended in a final volume of 5 ml DMEM/F12 + 5% (v/v) FCS. Fragments were transferred to a 10 cm dish and incubated in a 5% CO_2_ humidified 37 °C incubator for 30 min to allow fibroblast attachment. After shaking the dish, the fragment-containing media was transferred to a 15 ml tube and centrifuged at 1250 × *g* for 10 min. The pellet was resuspended in 2 ml PBS + 2% (v/v) FCS then 8 ml RBC lysis buffer (PBS + 0.8% (w/v) NH_4_Cl, 0.1 mM EDTA) was added. Fragments were incubated for 2 min then centrifuged at 1250 × *g* for 2 min. The supernatant was aspirated down to ~200 μl, and the fragments resuspended. Isolated organoids were mixed with Matrigel at ~10,000 cells/50 µl of matrix and seeded into 24-well plates. The Matrigel was polymerized at 37 °C for 30 min then overlayed with organoid media (DMEM/F12 + 2 mM L-glutamine, 100 units/ml penicillin, 1% (v/v) streptomycin, 20 ng/ml EGF, 20 ng/ml FGF2, 1x ITS, 10 μM Y27632 (only added for the first 4 days in culture), 5% (v/v) R-spondin1 conditioned medium) and cultured in a 5% CO_2_ humidified 37 °C incubator. Mammary organoids were maintained in culture by passaging every 2–3 weeks. Organoids were treated with trypsin, then centrifuged at 200 x *g* for 5 min. Mammary cells were resuspended in Matrigel (10,000 cells/50 µl) and seeded in 24-well plates as above without the addition of Y27632. Mammary organoid size was determined by measuring organoid area.

### Mammary organoid immunohistochemistry

Mammary organoids cultured in Matrigel were fixed by the addition of 500 μL 0.2% (w/v) paraformaldehyde in PBS. Organoids were fixed at 4 °C for 48 h with gentle agitation, then the Matrigel broken up by pipetting gently up and down with a P1000 pipette. Organoids were collected via centrifugation at 200 x *g* for 5 min. Organoid pellets were resuspended in 50-75 μL of Histogel and embedded in blocks of agarose. The Histogel was left on ice for 30 min to set then fixed in 10% formalin overnight, and paraffin-embedded.

5 µm sections from formalin-fixed, paraffin-embedded tissues/organoids were incubated in a 60 °C oven for 30 min. Tissue sections were dewaxed in three changes of xylene, then rehydrated in three changes of ethanol (2 min each). Heat-induced antigen retrieval was performed in a pressure cooker for 10 min in Novocastra Epitope Retrieval solution pH 9 or 10 mM citrate buffer pH 6. Sections were blocked in 1% BSA (w/v) in IHC-TBS (50 mM Tris-HCl, pH 7.5, 150 mM NaCl) for 1 h, then incubated with primary antibodies diluted in blocking buffer overnight at 4 °C. Sections were washed three times with IHC-TBS for 5 min each, then incubated with fluorescent-conjugated secondary antibodies or phalloidin and DAPI diluted in blocking buffer for 1 h in the dark at room temperature. Sections were washed three times for 5 min each, then coverslips mounted with Fluoromount G. Images were obtained using a Nikon invert confocal fluorescent microscope with NIS-elements version 4.13 software and analyzed using ImageJ software version 2.0.0.

### Droplet digital polymerase chain reaction

Droplet digital PCR utilized a Bio-Rad QX100 Droplet Digital PCR system. Reactions (20 µl) containing 1x ddPCR EvaGreen Supermix, 1.25 µl primers (Quantitect Primer Assay, Qiagen) and 500 ng cDNA were loaded into the middle wells of a droplet generator cartridge and 70 µl Droplet Generation Oil for EvaGreen (Bio-Rad) into the lower wells. Individual droplets were generated, then 40 µl of droplets were transferred to a 96-well PCR plate, sealed and subjected to thermal cycling according to the manufacturer’s instructions. Analysis of the completed reactions in individual reactions was detected using a droplet reader (Bio-Rad). The data was analyzed using QuantaSoft software (Bio-Rad) with the thresholds for detection set manually based on the results from the no-template control.

### Culture of human breast cancer cell lines

T47D (# HTB-133), MDA-MB-231 (# HTB-26), Hs578T, MCF-7, ZR-75-1, SKBR3 and BT549 human breast cancer cells and MCF-10A human mammary epithelial cells were purchased from American Type Culture Collections (Manassas, VA). SUM149PT and SUM185PE were purchased from Asterand Bioscience. T47D cells were cultured in RPMI supplemented with 10% (v/v) FCS, 2 mM L-glutamine, 100 units/ml penicillin, 1% (v/v) streptomycin, and 10 μg/ml insulin. MDA-MB-231 and MCF-7 cells were cultured in DMEM supplemented with 10% (v/v) FCS, 2 mM l-glutamine, 100 units/ml penicillin and 1% (v/v) streptomycin (and 10 μg/ml insulin for MCF-7 cells). Hs578T, ZR-75-1, SUM149PT, SUM185PE, BT549 and SKBR3 cells were cultured in RPMI supplemented with 10% (v/v) FCS, 2 mM L-glutamine, 100 units/ml penicillin, 1% (v/v) streptomycin, 10 μg/ml insulin and either 20 mM HEPES (Hs578T, ZR-75-1, SUM149PT, SUM185PE, BT549) or 1 mM pyruvate (SKBR3). MCF-10A cells were cultured in 5% (v/v) horse serum, 2 mM L-glutamine, 100 units/ml penicillin, 1% (v/v) streptomycin, 0.5 μg/ml hydrocortisone, 100 ng/ml cholera toxin, 10 μg/ml insulin and 5 ng/ml EGF. Cell lines were maintained in a 5% CO_2_ humidified 37 °C incubator. Cell lines were not authenticated, but were maintained for less than 2 months. Cell lines were tested by PCR to confirm the absence of mycoplasma contamination.

For serum stimulation, cells were incubated overnight in phenol red-free DMEM (MDA-MB-231, MCF-7) or RPMI (T47D, Hs578T, ZR-75-1, SKBR3, SUM149PT, SUM185PE, BT549) without FCS. The following day, complete media containing 10% FCS was added for 10 mins. For EGF or insulin stimulation, cells were washed once with PBS, then incubated overnight with DMEM (MDA-MB-231 cells) or phenol red-free RPMI (T47D, Hs578T cells) without FCS. The following day, EGF (100 ng/ml) diluted in DMEM or phenol red-free RPMI without FCS was added for the indicated time points.

### Generation of stable *PIPP* and *PTEN* knockdown T47D, MDA-MB-231 and Hs578T cells

T47D, MDA-MB-231 or Hs578T cells were transduced with Sigma MISSION lentiviral particles carrying the pLKO.1-puro plasmid encoding either a non-targeting shRNA or an shRNA sequence targeting human *PIPP* (CCTGGGCTACTATAGTCACAA) (Sigma-Aldrich, USA) together with Sigma MISSION lentiviral particles carrying the pLKO.1-neo plasmid encoding either a non-targeting shRNA or an shRNA sequence targeting human *PTEN* (AGGCGCTATGTGTATTATTAT) at a multiplicity of infection (MOI) of 1 in growth media supplemented with 8 µg/ml hexadimethrine bromide overnight. For selection, transduced cells were cultivated in culture medium supplemented with puromycin (T47D cells: 0.3 µg/ml, MDA-MB-231 and Hs578T cells: 1 µg/ml) and G418 (T47D cells: 0.4 mg/ml, MDA-MB-231 and Hs578T cells: 1 mg/ml) and passaged for 10 days before being transferred to maintenance media containing (T47D cells: 0.25 µg/ml puromycin and 0.25 mg/ml G418, MDA-MB-231 and Hs578T cells: 0.5 µg/ml puromycin and 0.5 mg/ml G418). At least 2 days post-selection, total RNA was isolated from transduced cells using the RNeasy RNA isolation kit (Qiagen, USA) to test for *PIPP* and *PTEN* mRNA expression by quantitative real-time PCR.

### Quantitative real-time polymerase chain reaction (PCR)

Total RNA was extracted from human T47D, MDA-MB-231 and Hs578T breast cancer cell lines using either the RNeasy RNA isolation kit (Qiagen, USA) or the Isolate II RNA extraction kit (Bioline), according to the manufacturer’s instructions. Two-step quantitative real-time PCR was performed using the iScript gDNA clear cDNA synthesis kit (Bio-Rad) and the Quantitect SYBR Green PCR Kit (Qiagen) or the AffinityScript qPCR cDNA synthesis kit and the Brilliant II SYBR Green qPCR Master-mix kit (Stratagene-Agilent Technologies), according to the manufacturer’s instructions. Reactions were subjected to thermocycling using a RotorGene 6000 Real Time PCR machine (Qiagen) and analyzed with RotorGene Q Series 2.3.5 software. The relative expression of the gene of interest, compared to *GAPDH*, was calculated using the ΔΔCt method, as previously described^74^.

### Bromo-2-deoxyuridine (BrdU) cell proliferation assay

T47D or MDA-MB-231 cells expressing control, *PIPP* and/or *PTEN*-specific shRNAs were grown on glass coverslips, and serum starved for 24 h (T47D) or 43 h (MDA-MB-231). Cells were treated with 10 nM BrdU for 45 min (T47D) or 5 h (MDA-MB-231) before fixation with BrdU fixative for 20 min at –20 °C. BrdU incorporation was assessed using a BrdU-labeling and detection kit I (Roche, Germany) according to the manufacturer’s instructions. Cell nuclei were co-stained with DAPI. Random fields/coverslip (x5-10) were imaged using a Nikon C1 confocal microscope with 20x objective (Monash Micro Imaging, Monash University, Australia). The percentage of BrdU-positive nuclei was determined using ImageJ analysis software version 2.0.0.

For rescue studies, T47D cells were transiently transfected with plasmids encoding either HA-vector or HA-PIPP^23^ using Lipofectamine 3000 according to the manufacturer’s instructions and cultured for 24 h then serum starved for 24 h. Cells were treated with 10 nM BrdU for 45 min before fixation with 4% paraformaldehyde in PBS for 20 min. Cells were washed three times with PBS, then permeabilized in 0.1% (v/v) Triton X-100 in PBS for 5 min and blocked in 1% BSA in PBS for 15 min. HA antibodies were diluted in block and added for 1 h then cells were washed 3 times in PBS. Alexa Fluor® secondary antibodies (Life Technologies) were diluted in the block and added for 1 h. Cells were washed three times with PBS, then incubated with 2 N HCl in PBS for 30 min. After washing three times with PBS BrdU incorporation was detected and assessed as above.

### Clonogenic assay

For colony growth assays, 800 cells per cell line were seeded into 6-well dishes and cultured for 8 d then fixed with 6% (v/v) glutaraldehyde or 10% neutral buffered formalin for 30 min and stained with DiffQuick (Lab Aids P/L, Australia). Colonies were imaged with a Leica M165C microscope (2.5× magnification) and a Leica DFC295 camera with a KL1500LCD light box or an Olympus MVX10 microscope (0.63x magnification) (4 fields/cell line).

### Anchorage-independent cell growth assay

2 × 10^3^ control, *PIPP* and/or *PTEN* shRNA T47D cells were suspended in 5 ml DMEM, 10% FCS, 0.3% agar and plated over 2 ml DMEM, 10% FCS, 0.7% agar in 6-well dishes in triplicate. Cells were incubated at 37 °C for 4 weeks. Wells were imaged using a Leica M165C microscope (1.25× magnification) and a Leica DFC295 camera with a KL1500LCD light box (4 images/well). Colony number and size was quantified using ImageJ analysis software.

### Cell migration assay

5 × 10^4^ control, *PIPP* and/or *PTEN* shRNA MDA-MB-231 cells were seeded in the top chamber of a Transwell (Corning, NY) in triplicate in serum-free media and allowed to migrate towards DMEM media supplemented with 10% FCS in the bottom chamber for 3 h. Non-migrated cells were removed from the upper chamber surface with a cotton swab and cells which had migrated to the underside of the chamber were fixed and stained using the DiffQuick Staining Kit (Lab Aids P/L, Australia). Cells were imaged using a 20× objective on an Olympus CKX41 inverted light microscope (Biochemistry Imaging Facility, Monash University, Australia). The average number of migrated cells was scored from 12 fields/Transwell using Image J analysis software version 2.0.0.

### Immunoblotting

Cell lysates were prepared by washing cells once with cold TBS (20 mM Tris-HCl pH 7.4, 150 mM NaCl) on ice, followed by direct cell lysis in 40 mM Tris pH 6.8, 4% (w/v) SDS, 20% (v/v) glycerol, 0.0002% (w/v) bromophenol blue, 50 mM DTT. Alternatively, cells were washed in cold PBS, scraped in lysis buffer (20 mM Tris-HCl, pH 7.4, 150 mM NaCl with 1x complete protease inhibitor (Roche) and 1x PhosSTOP (Roche)) and cleared by centrifugation. Protein concentration was determined with a DC Protein Assay (BioRad) then standard Laemmli-Buffer with 5% final concentration of β-mercaptoethanol was added to samples. Lysates were boiled for 5 min at 100 °C, and proteins were separated by 10% SDS-PAGE at 100–200 V for 1–1.5 h. Proteins were transferred to PVDF by electrophoresis at 250 mA for 1 h. Membranes were blocked in SDS-PAGE blocking buffer (5% skim milk in TBS) on a rocker at room temperature for 1 h then incubated with primary antibodies diluted in TSB-T (TBS, 0.1% (v/v) Tween-20) overnight at 4 °C on a rocker. Membranes were washed three times with TBS-T while rocking for 10 min each. Secondary HRP-conjugated antibodies diluted in TBS-T were added for 1 h at room temperature, then membranes were washed three times in TBS-T on a rocker. Membranes were incubated in ECL Plus for 1 min, then exposed to X-ray film in a dark room and developed using a Fuji processor. Immunoblots were re-probed with antibodies specific for the housekeeping protein GAPDH or pan-ACTIN.

### TissueScan breast cancer cDNA arrays (I–IV)

*PTEN* mRNA expression was measured in TissueScan Breast Cancer cDNA Arrays I, II, III and IV (OriGene, BCRT101, BCRT102, BCRT103, and BCRT104) which contained 176 breast cancers and 16 tumor-adjacent “normal” breast tissues. Relative transcript levels were determined using *PTEN* and *B-ACTIN* primers (OriGene) in an MX3000p qPCR system (Stratagene) according to the manufacturer’s instructions. *PTEN* mRNA expression was normalized to *β-ACTIN* using the ΔΔCt method^74^. *PTEN* mRNA expression in the breast cancer samples was calculated relative to the mean of the normal tissue samples. *PIPP* mRNA expression in the TissueScan Breast Cancer cDNA Arrays I, II, III and IV has been previously reported^23^. Statistical significance was determined by a Mann–Whitney test assuming non-Gaussian distribution for two groups or a Kruskal–Wallis test assuming non-Gaussian distribution for more than two groups.

### Statistics and reproducibility

All statistical analyses were performed using GraphPad Prism 10, and p < 0.05 was considered statistically significant. Unless otherwise stated in the figure legend, p-values were determined by using an unpaired Student’s *t* test (two-tailed) for analysis of two groups or one-way ANOVA with Tukey’s correction for more than two groups. All experimental values are presented as means ± SEM unless otherwise stated. All experiments were performed independently at least three times to ensure reproducibility except Supplementary Fig. S2F, G which was performed twice. The exact number of biological and technical replicates is indicated in the corresponding figure legends.

### Reporting summary

Further information on research design is available in the Nature Portfolio Reporting Summary linked to this article.

## Supplementary information

Supplementary information

Description of Additional Supplementary Files

Supplementary Data 1

Reporting summary

## Data Availability

All data necessary to evaluate the conclusions in this paper are included in the manuscript and/or the Supplementary files. The source data behind the graphs are provided in the Supplementary Data 1 file. Images of uncropped blots are provided in Supplementary Information as Fig. S7. Gene expression data from the METABRIC and TCGA datasets are available in cBioPortal. Any additional data related to this study is available upon reasonable request from the corresponding author.
