# Supplementary material for: Non-redundant roles of the phosphoinositide phosphatases PTEN and PIPP in PI3K/AKT signaling in breast cancer
Source: Commun Biol. 2025 Dec 17;9:96. doi: 10.1038/s42003-025-09364-2 (PMC12827249; doi:10.1038/s42003-025-09364-2)
Supplement: Supplementary file 1 — Supplementary information [file 42003_2025_9364_MOESM1_ESM.pdf]

Non-redundant roles of the phosphoinositide phosphatases PTEN and PIPP in PI3K/AKT signaling in breast cancer.

Lisa M. Ooms, Daniel T. Ferguson, Samuel J. Rodgers, Karmanpreet K. Sukhija, Emily I. Jones, Mariah P. Csolle, Hon Yan Kelvin Yip, Roger J. Daly, Tony Tiganis, Catriona A. McLean, Antonella Papa, Christina A. Mitchell.

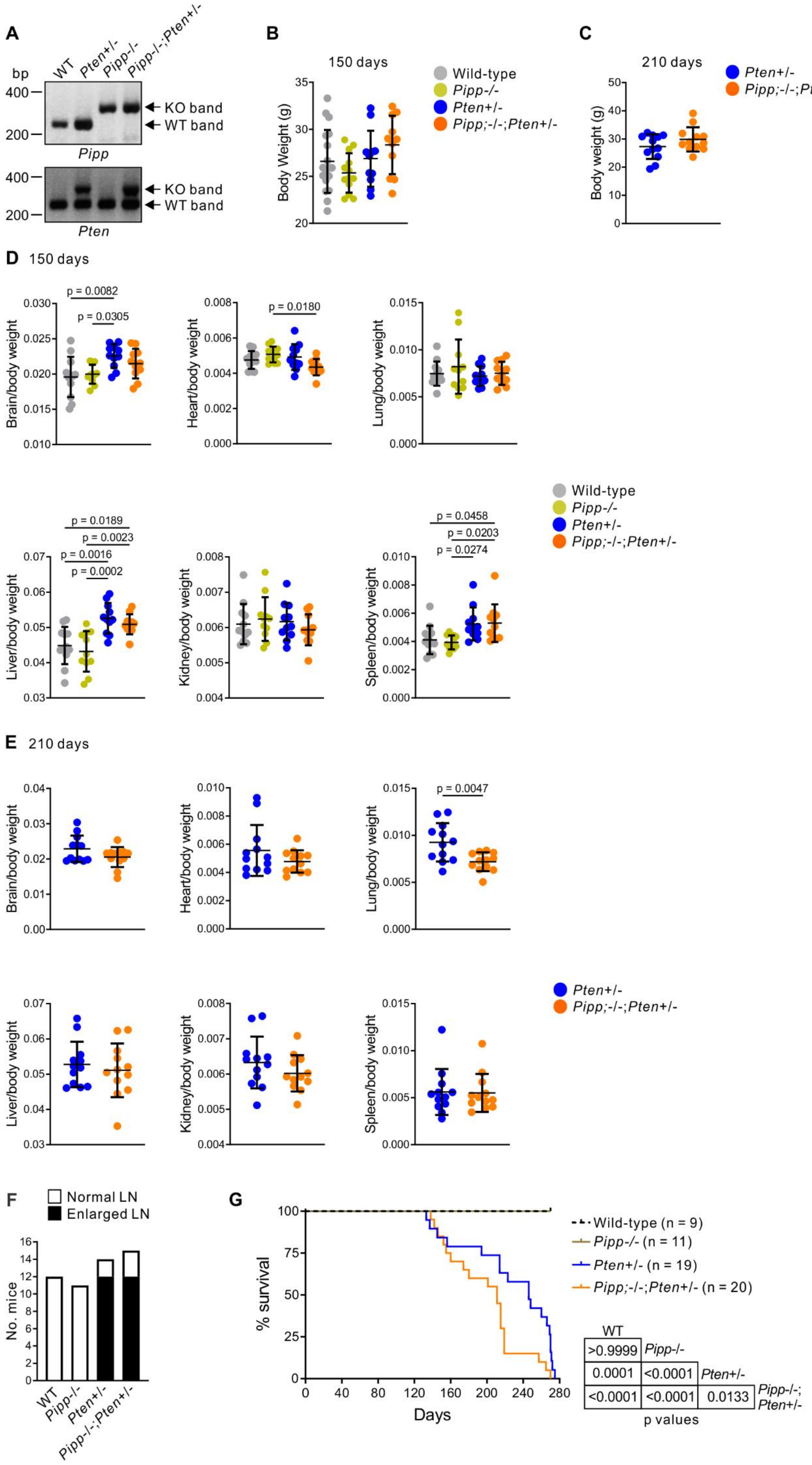
A
bp
WT
Pten+/-
Pipp-/-
Pipp-/-;Pten+/-
400
200
KO band
WT band
Pipp
Pten
B
150 days
Body Weight (g)
Wild-type
Pipp-/-
Pten+/-
Pipp;-/-;Pten+/-
C
210 days
Body weight (g)
Pten+/-
Pipp;-/-;Pten+/-
D 150 days
Brain/body weight
p = 0.0082
p = 0.0305
Heart/body weight
p = 0.0180
Lung/body weight
Liver/body weight
p = 0.0189
p = 0.0023
p = 0.0016
p = 0.0002
Kidney/body weight
Spleen/body weight
p = 0.0458
p = 0.0203
p = 0.0274
Wild-type
Pipp-/-
Pten+/-
Pipp;-/-;Pten+/-
E 210 days
Brain/body weight
Heart/body weight
Lung/body weight
p = 0.0047
Liver/body weight
Kidney/body weight
Spleen/body weight
Pten+/-
Pipp;-/-;Pten+/-
F
Normal LN
Enlarged LN
No. mice
WT
Pipp-/-
Pten+/-
Pipp-/-;Pten+/-
G
% survival
Days
Wild-type (n = 9)
Pipp-/- (n = 11)
Pten+/- (n = 19)
Pipp;-/-;Pten+/- (n = 20)
WT
>0.9999 Pipp-/-
0.0001 <0.0001 Pten+/-
<0.0001 <0.0001 0.0133 Pipp-/-; Pten+/-
p values

Supplementary Fig. S1: *Pipp* ablation reduces survival of *Pten*$^{+/-}$ mice.

A. PCR of genomic DNA showing the expected 247 bp (wild-type) DNA fragment in the *Pten*$^{+/+}$ mice and the 247 bp (wild-type) and 350 bp (knockout) DNA fragment in the *Pten*$^{+/-}$ mice and DNA fragments of 255 bp and 383 bp, corresponding to wild-type *Pipp* and the targeted allele, respectively.

B-C. Data represent body weight of 150 d-old wild-type (n = 18), *Pipp*$^{-/-}$ (n = 13), *Pten*$^{+/-}$ (n = 11) and *Pipp*$^{-/-}$*;Pten*$^{+/-}$ (n = 11) mice (**B**) and 210 d-old *Pten*$^{+/-}$ (n = 12) and *Pipp*$^{-/-}$*;Pten*$^{+/-}$ (n = 12) mice (**C**) ± SEM.

D. Data represent organ weight to body weight ratios in 150 d-old wild-type (n = 12), *Pipp*$^{-/-}$ (n = 11), *Pten*$^{+/-}$ (n = 11) and *Pipp*$^{-/-}$*;Pten*$^{+/-}$ (n = 11) mice ± SEM.

E. Data represent organ weight to body weight ratios in 210 d-old *Pten*$^{+/-}$ (n = 12) and *Pipp*$^{-/-}$*;Pten*$^{+/-}$ (n = 12) mice ± SEM.

F. Data represent the number of wild-type (n = 12), *Pipp*$^{-/-}$ (n = 11), *Pten*$^{+/-}$ (n = 14) and *Pipp*$^{-/-}$*;Pten*$^{+/-}$ (n = 15) mice with enlarged lymph nodes.

G. Kaplan-Meier survival curves of wild-type (n = 12), *Pipp*$^{-/-}$ (n = 11), *Pten*$^{+/-}$ (n = 14) and *Pipp*$^{-/-}$*;Pten*$^{+/-}$ (n = 15) mice showing *Pipp* ablation in *Pten*$^{+/-}$ mice significantly reduces time to endpoint due to lymphadenopathy.

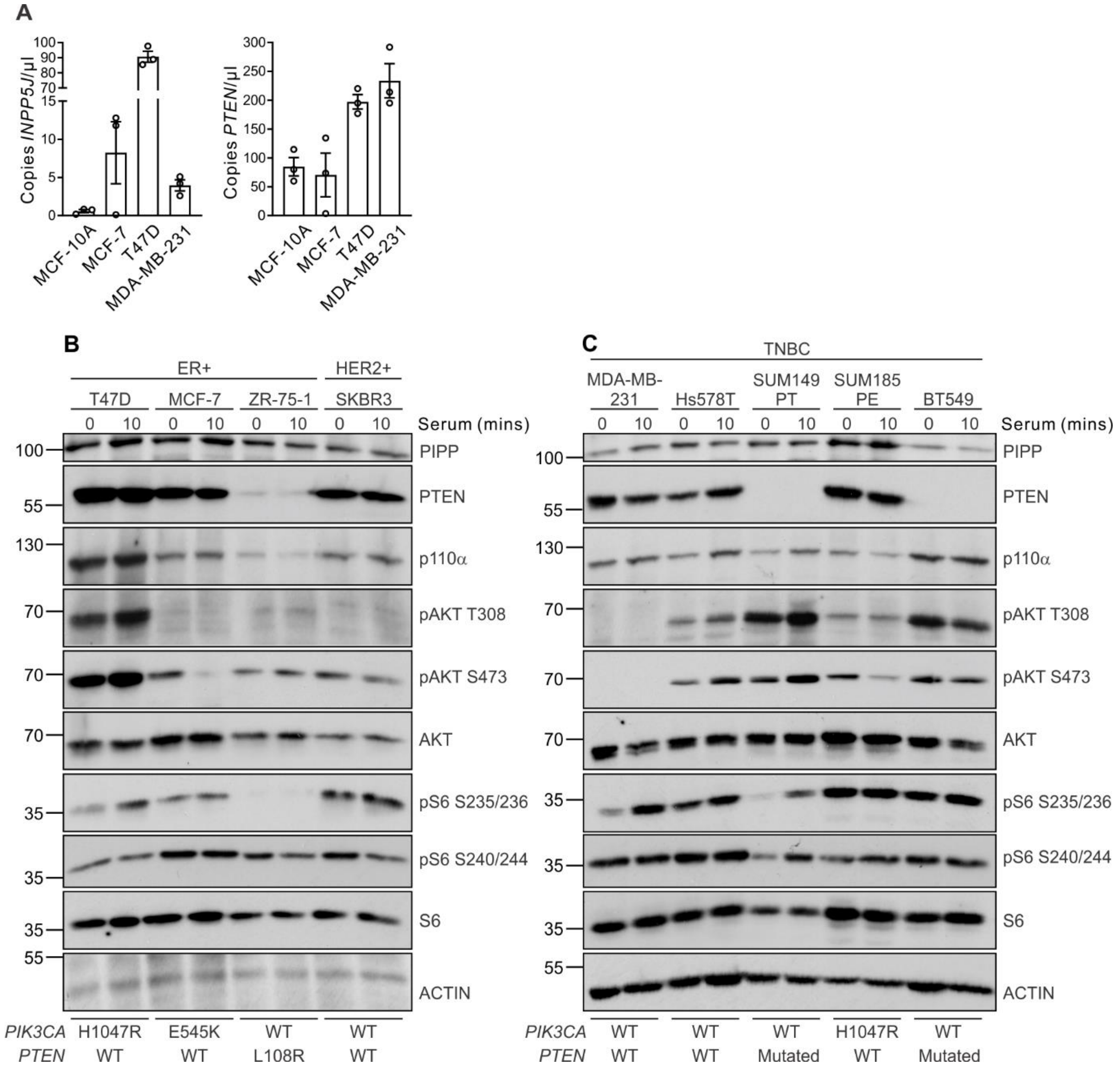

Supplementary Fig. S2: Characterization of PI3K/AKT signaling in human breast cancer cell lines.

A. Absolute *PIPP* and *PTEN* expression was quantified by droplet digital PCR in mRNA extracted from MCF-10A (non-transformed mammary epithelial cell line), MCF-7 (ER+), T47D (ER+) and MDA-MB-231 (triple-negative) cell lines. Bars represent mean *PIPP* or *PTEN* expression ± SEM (n = 4 independent experiments).

B-C. ER+ (T47D, MCF-7, ZR-75-1), HER2+ (SKBR3) (**B**) or triple negative breast cancer (MDA-MB-231, Hs578T, SUM149PT, SUM185PE, BT549) (**C**) cell lines were serum starved overnight then stimulated with media containing 10% fetal calf serum. Cells were lysed and immunoblotted

with PIPP, PTEN, p110α, pAKT Thr308, pAKT Ser473, AKT, pS6 Ser235/236, pS6 Ser240/244, S6 or ACTIN antibodies as a loading control.

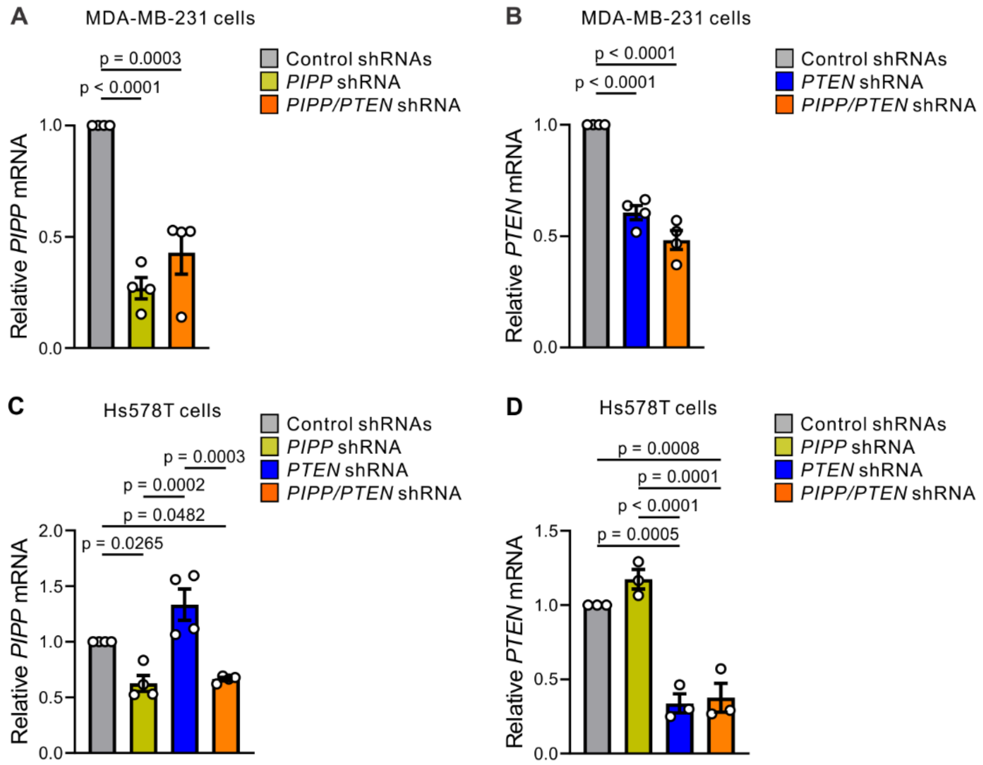

Supplementary Fig. S3: Generation of *PIPP, PTEN* and *PIPP/PTEN* knockdown MDA-MB-231 and Hs578T cells.

A-D. MDA-MB-231 (**A, B**) or Hs578T (**C, D**) cells were transduced with lentiviral particles encoding non-target control, *PIPP, PTEN* or *PIPP/PTEN* shRNA. RNA was extracted and subjected to quantitative real-time PCR using primers for *PIPP* (**A, C**) or *PTEN* (**B, D**). Expression was normalized to *GAPDH*. Expression was quantified from 3 (**D**) or 4 (**C**) independent experiments using the ΔΔCt method. Data represent mean transcript levels ± SEM, relative to MDA-MB-231 or Hs578T cells expressing control shRNAs which were arbitrarily assigned a value of 1.

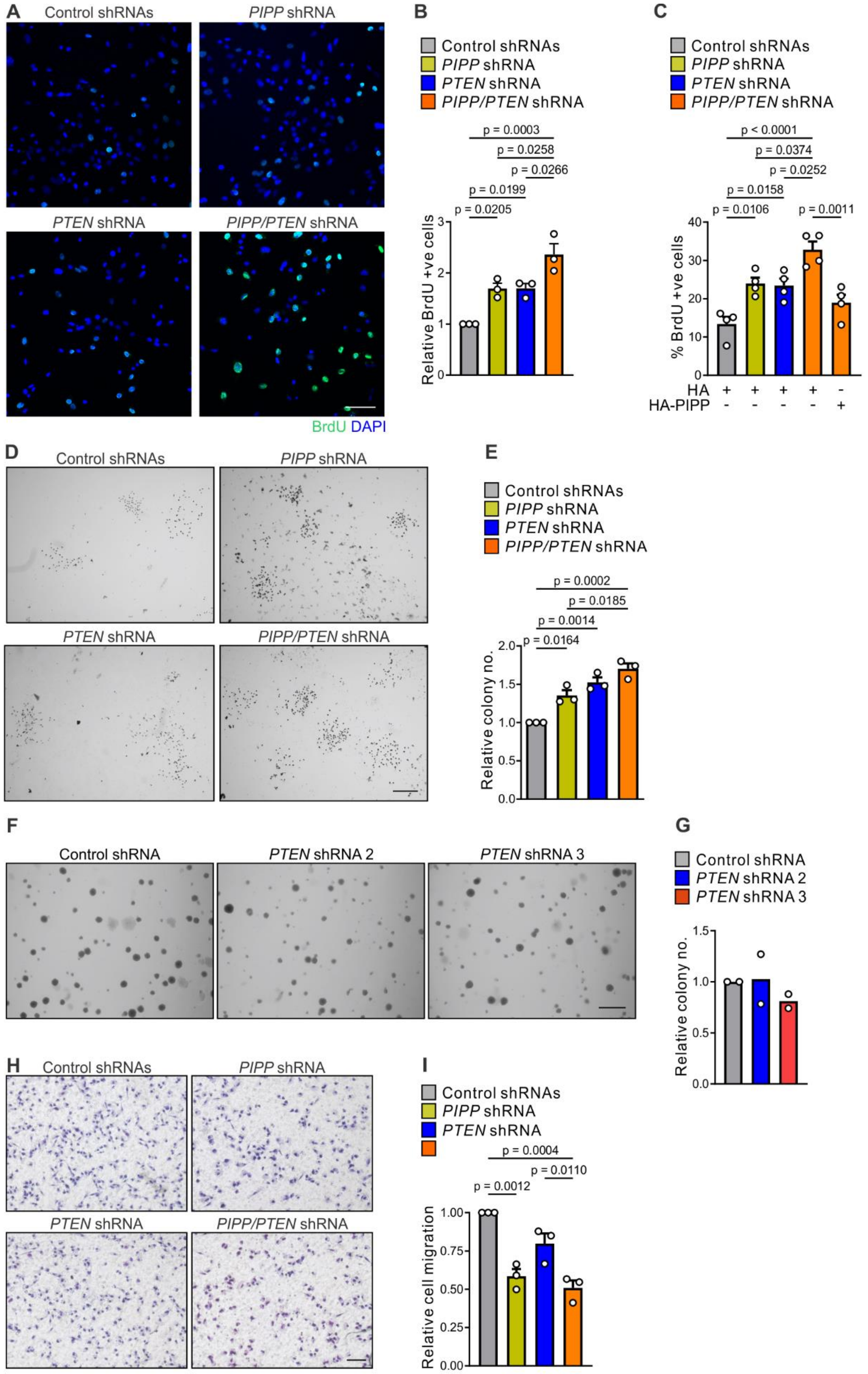
A
Control shRNAs
PIPP shRNA
PTEN shRNA
PIPP/PTEN shRNA
BrdU DAPI
B
Control shRNAs
PIPP shRNA
PTEN shRNA
PIPP/PTEN shRNA
p = 0.0003
p = 0.0258
p = 0.0266
p = 0.0199
p = 0.0205
Relative BrdU +ve cells
C
p < 0.0001
p = 0.0374
p = 0.0252
p = 0.0158
p = 0.0106
p = 0.0011
% BrdU +ve cells
HA
HA-PIPP
D
E
p = 0.0002
p = 0.0185
p = 0.0014
p = 0.0164
Relative colony no.
F
Control shRNA
PTEN shRNA 2
PTEN shRNA 3
G
H
I
p = 0.0004
p = 0.0110
p = 0.0012
Relative cell migration

Supplementary Fig. S4: Co-shRNA knockdown of *PIPP* and *PTEN* enhances MDA-MB-231 breast cancer cell proliferation.

A-B. MDA-MB-231 cells expressing control, *PIPP*, *PTEN* or *PIPP/PTEN* shRNA were serum-starved for 43 h, incubated with BrdU for 5 h then fixed and stained with BrdU antibodies and DAPI (**A**). Data represent mean percentage of BrdU-positive cells ± SEM (n = 3 independent experiments, >300 cells/experiment) (**B**).

C. T47D cells expressing control, *PIPP*, *PTEN* or *PIPP/PTEN* shRNA were transiently transfected with constructs encoding shRNA-resistant HA-PIPP or HA-vector alone. Cells were serum-starved for 24 h, incubated with BrdU for 45 min then fixed and stained with HA and BrdU antibodies and DAPI. Data represent mean percentage of BrdU-positive cells ± SEM (n = 4 independent experiments, >78 cells/experiment).

D-E. MDA-MB-231 cells stably transduced with control, *PIPP*, *PTEN* or *PIPP/PTEN* shRNA were seeded into 6-well dishes (800 cells/cell line), cultured for 1 week then fixed, stained with DiffQuick and imaged via light microscopy (**D**). Data represent the number of colonies ± SEM relative to control shRNA-expressing MDA-MB-231 cells which were arbitrarily assigned a value of 1 (n = 3 independent experiments) (**E**).

F-G. T47D cells stably transduced with control or one of two different *PTEN* shRNAs were suspended in 0.3% agar and cultured for 4 weeks (**F**). Data represent the relative number of colonies (**G**) (n = 2 independent experiments in triplicate, >500 colonies/experiment).

H-I. Migration of MDA-MB-231 cells stably transduced with control, *PIPP*, *PTEN* or *PIPP/PTEN* shRNA towards a serum gradient was determined using a Transwell assay (**H**). Data represent the number of migrated cells ± SEM relative to control shRNA-expressing MDA-MB-231 cells which were arbitrarily assigned a value of 1 (n = 3 independent experiments) (**I**).

Scale bars, 100 µm (A, H), 2 mm (D, F).

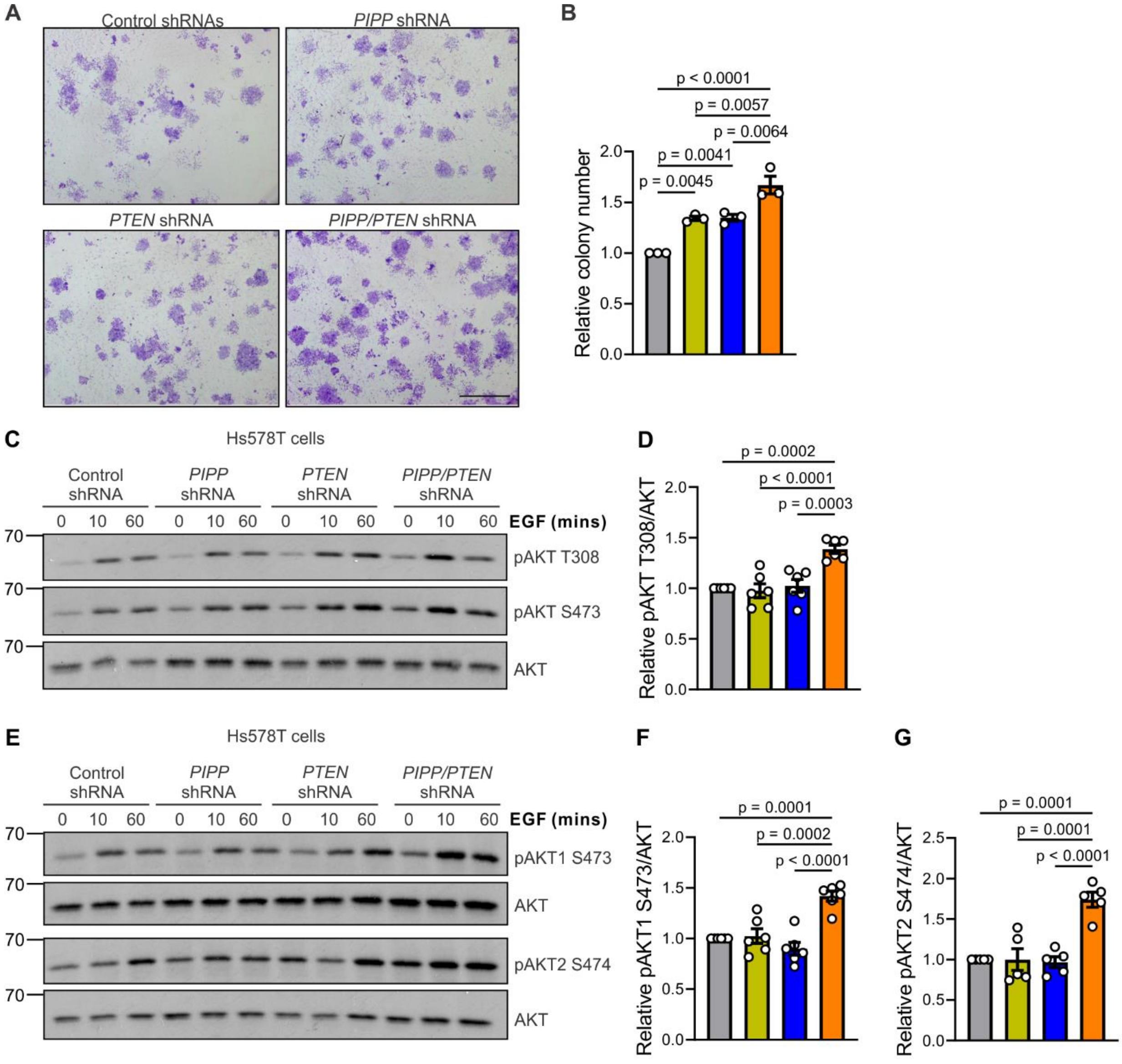

Supplementary Fig. S5: Co-shRNA knockdown of *PIPP* and *PTEN* enhances Hs578T cell proliferation and AKT signaling.

A-B. Hs578T cells stably transduced with control, *PIPP*, *PTEN* or *PIPP/PTEN* shRNA were seeded into 6-well dishes (800 cells/cell line), cultured for 1 week then fixed, stained with DiffQuick and imaged via light microscopy (**A**). Data represent the number of colonies ± SEM relative to control shRNA-expressing Hs578T cells which were arbitrarily assigned a value of 1 (n = 3 independent experiments) (**B**).

C-D. Hs578T *PIPP*, *PTEN, PIPP/PTEN* or control shRNA cells were serum starved overnight, stimulated with 100 ng/ml EGF for the indicated times then lysed and immunoblotted with pAKT

Thr308, pAKT Ser473, or AKT antibodies (**C**). Data represent mean pAKT Thr308 (n = 6 independent experiments) (**C**) relative to AKT ± SEM.

E-G. Hs578T *PIPP*, *PTEN, PIPP/PTEN* or control shRNA cells were serum starved overnight, stimulated with 100 ng/ml EGF then lysed and immunoblotted with pAKT1 Ser473, pAKT2 Ser474 or AKT antibodies (**E**). Data represent mean pAKT1 Ser473 (n = 6) (**F**) and pAKT2 Ser474 (n = 5 independent experiments) (**G**) relative to AKT ± SEM.

Scale bar, 500 μm (A).

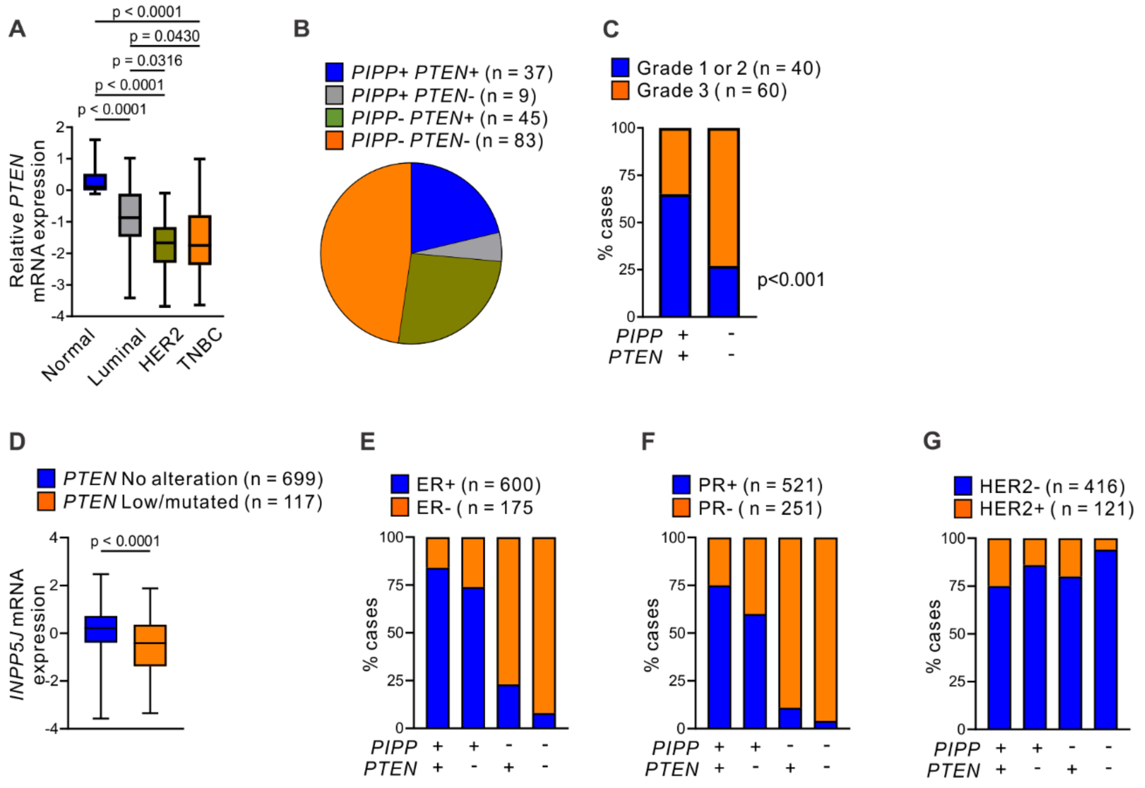

Supplementary Fig. S6: Low *PIPP/PTEN* mRNA expression occurs in a subset of breast cancers.

A. Normalized *PTEN* mRNA expression was determined by qPCR using TissueScan Breast Cancer Arrays I-IV with *PTEN* and β-actin primers. The data are displayed as box and whiskers on a log scale. The centre line indicates the median; the box extends from the 25th to 75th percentiles and the whiskers extend from the minimum to maximum values. *PTEN* mRNA expression was correlated with breast cancer subtype (133 breast cancer cases, 16 normal (non-cancerous tissue adjacent to the breast tumor) cases). p values were determined using one-way ANOVA with Tukey's post-hoc test.

B. *PIPP* mRNA expression was correlated with *PTEN* mRNA expression in TissueScan Breast Cancer Arrays I-IV. Low expression (−) represents >2-fold reduction in expression relative to normal breast tissue.

C. Breast cancer cases in TissueScan Breast Cancer Arrays I-IV were scored for normal (*PIPP+/PTEN+*) versus low (*PIPP-/PTEN-*) *PIPP* and *PTEN* mRNA expression in Grade 1 or 2 and

Grade 3 tumors (100 cases). Significance was determined using a two-sided Fisher's exact test (p<0.001).

D. *PIPP* mRNA expression was correlated with *PTEN* alterations in the TCGA dataset. *PIPP* expression was correlated with unaltered *PTEN* versus altered *PTEN* (mutated and/or low expression). The data are displayed as box and whiskers. The centre line indicates the median; the box extends from the 25th to 75th percentiles and the whiskers extend from the minimum to maximum values. p values were determined using an unpaired t test.

E-G. Breast cancer cases in the TCGA dataset were scored for reduced *PIPP* expression and/or *PTEN* expression (Z-score threshold of <1.5 relative to all breast cancers in the cohort) and/or *PTEN* mutation and correlated with ER (**E**), PR (**F**) or HER2 (**G**).

Fig. 6A

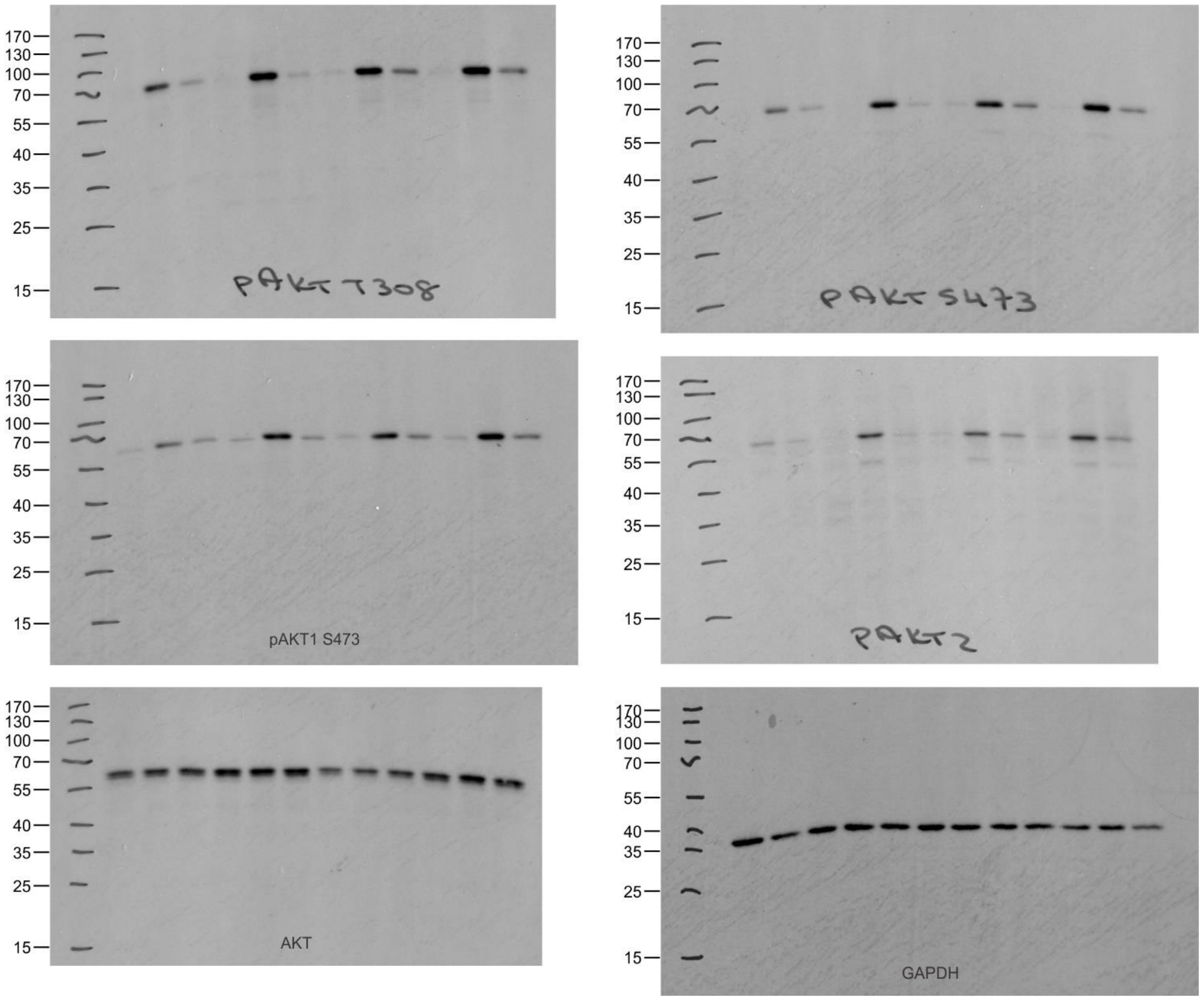
170
130
100
70
55
40
35
25
15
pAKT T308
pAKT S473
pAKT1 S473
pAKT2
AKT
GAPDH

Fig. 6F

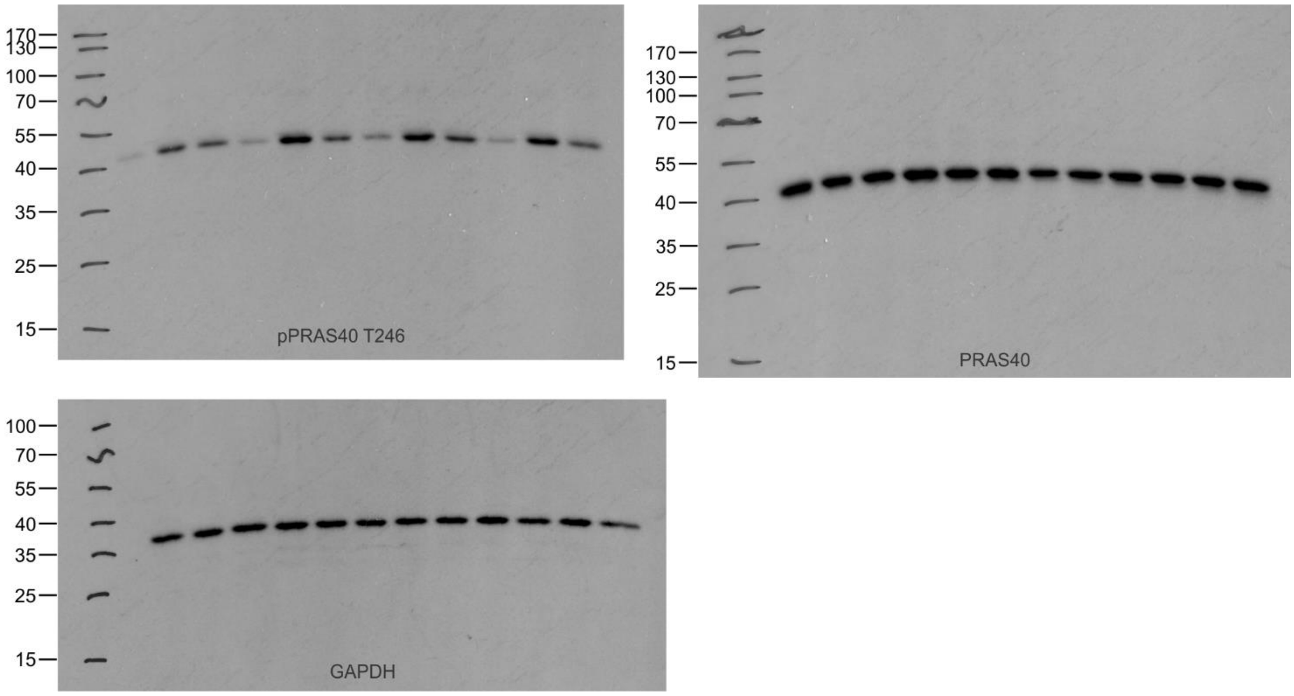

Supplementary Fig. S2B

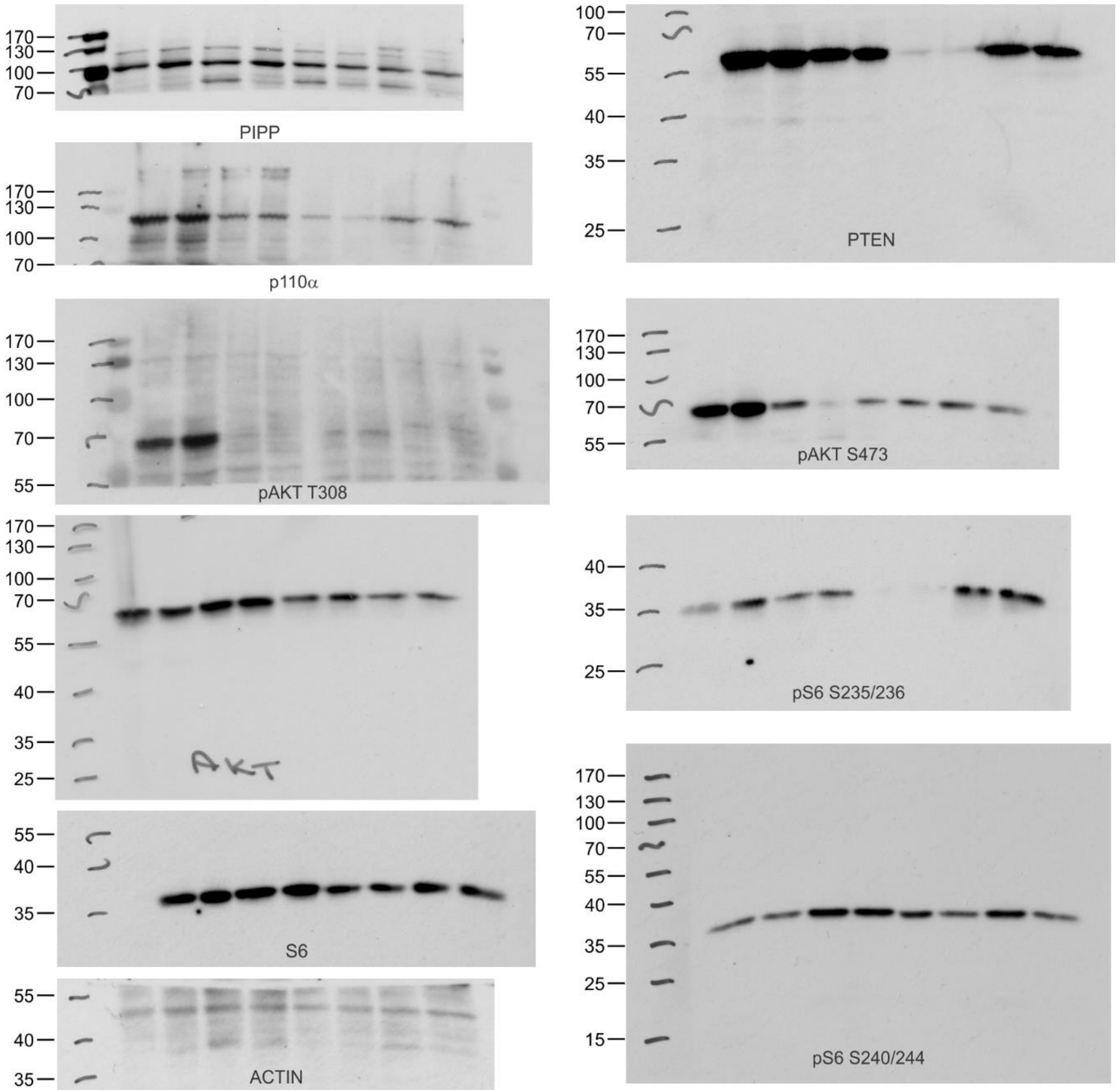

Supplementary Fig. S2C

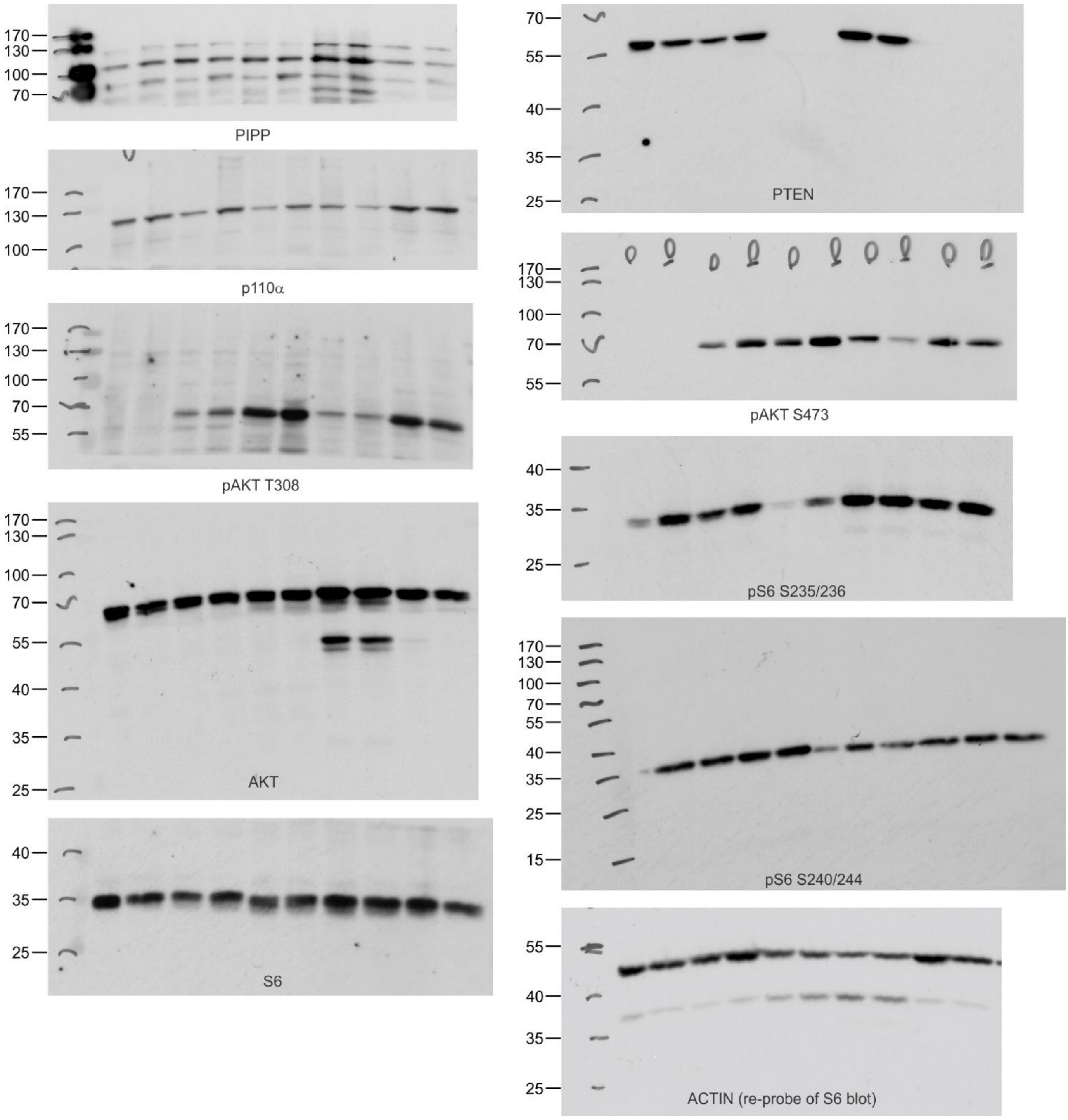

Supplementary Fig. S5C

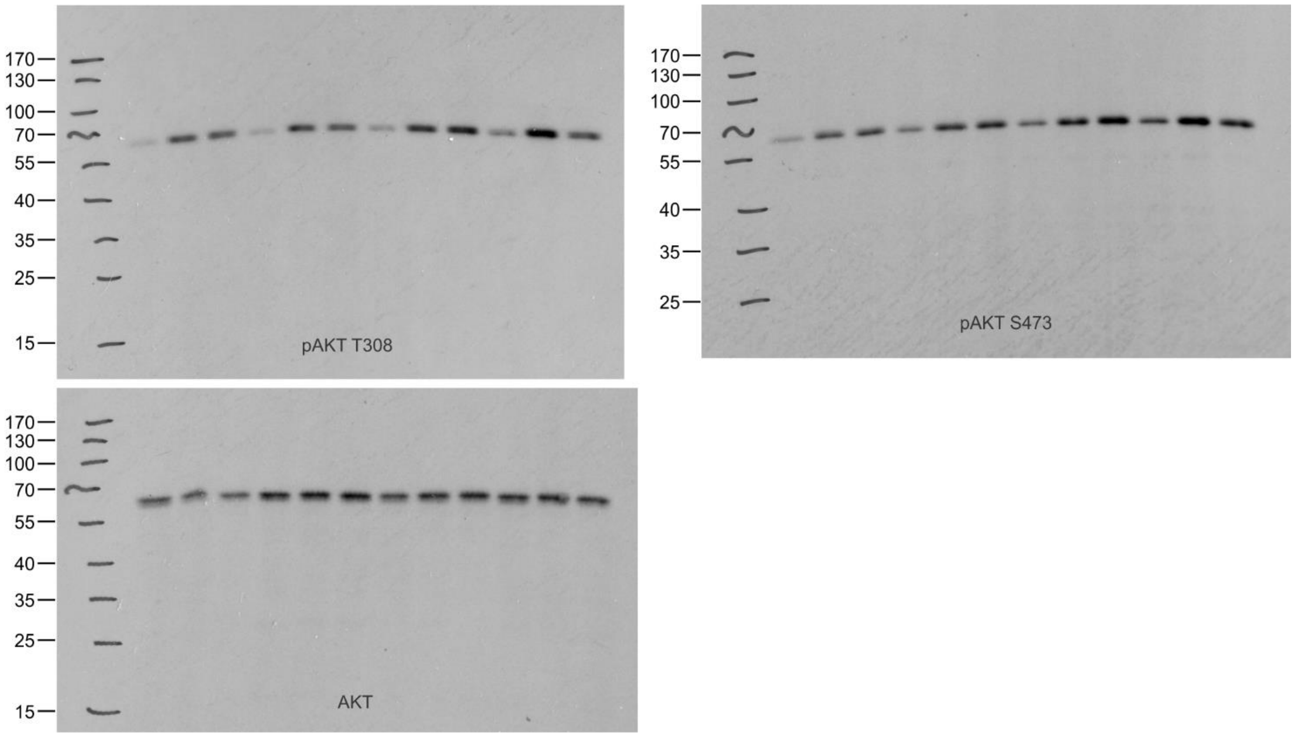

Supplementary Fig. S5E

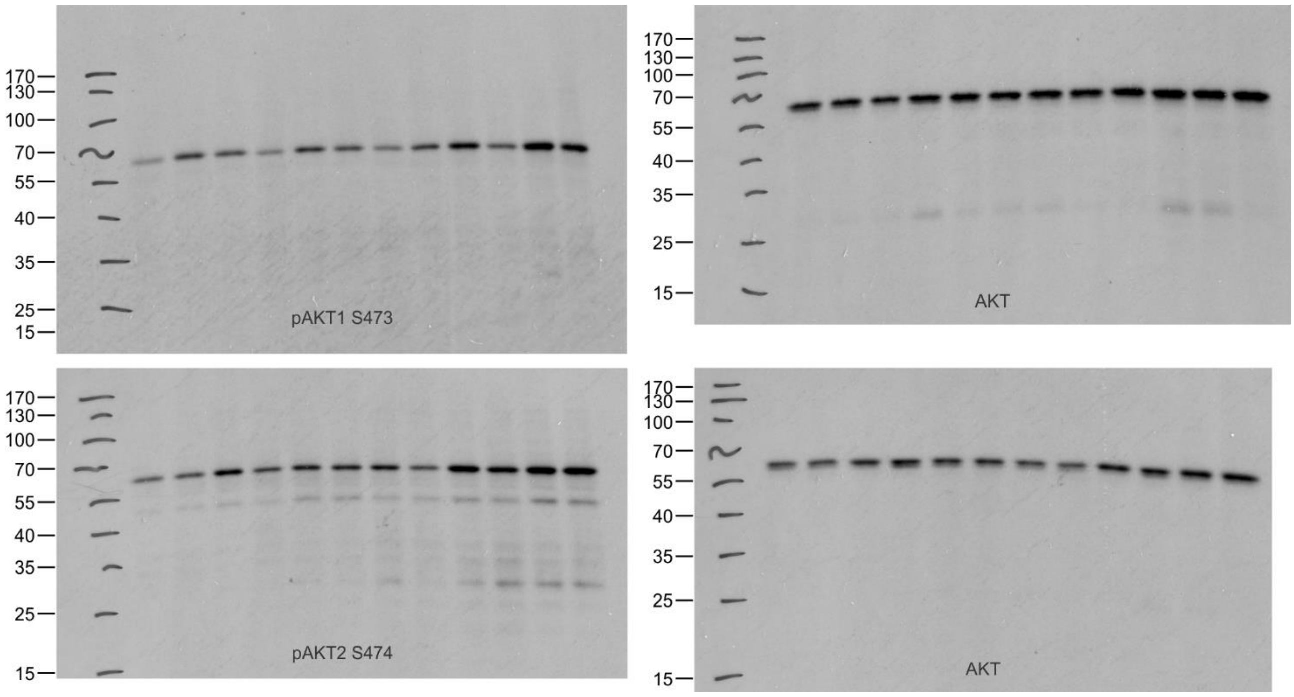

Supplementary Fig. S7: Uncropped immunoblots for figures.
