# Supplementary material for: Non-redundant roles of the phosphoinositide phosphatases PTEN and PIPP in PI3K/AKT signaling in breast cancer
Source: Commun Biol. 2025 Dec 17;9:96. doi: 10.1038/s42003-025-09364-2 (PMC12827249; doi:10.1038/s42003-025-09364-2)
Supplement: Supplementary file 2 — Description of Additional Supplementary Files [file 42003_2025_9364_MOESM2_ESM.pdf]

Description of Additional Supplementary files

File name: Supplementary Data 1

Description: The source data behind the graphs are provided in the Supplementary Data 1
