# Supplementary material for: Non-redundant roles of the phosphoinositide phosphatases PTEN and PIPP in PI3K/AKT signaling in breast cancer
Source: Commun Biol. 2025 Dec 17;9:96. doi: 10.1038/s42003-025-09364-2 (PMC12827249; doi:10.1038/s42003-025-09364-2)
Supplement: Supplementary file 4 — Reporting summary [file 42003_2025_9364_MOESM4_ESM.pdf]

nature portfolio

Corresponding author(s): Christina Mitchell

Last updated by author(s): Nov 19, 2025

Reporting Summary

Nature Portfolio wishes to improve the reproducibility of the work that we publish. This form provides structure for consistency and transparency in reporting. For further information on Nature Portfolio policies, see our Editorial Policies and the Editorial Policy Checklist.

Statistics

For all statistical analyses, confirm that the following items are present in the figure legend, table legend, main text, or Methods section.

n/a	Confirmed	
☐	☒	The exact sample size (*n*) for each experimental group/condition, given as a discrete number and unit of measurement
☐	☒	A statement on whether measurements were taken from distinct samples or whether the same sample was measured repeatedly
☐	☒	The statistical test(s) used AND whether they are one- or two-sided<br>*Only common tests should be described solely by name; describe more complex techniques in the Methods section.*
☒	☐	A description of all covariates tested
☐	☒	A description of any assumptions or corrections, such as tests of normality and adjustment for multiple comparisons
☐	☒	A full description of the statistical parameters including central tendency (e.g. means) or other basic estimates (e.g. regression coefficient) AND variation (e.g. standard deviation) or associated estimates of uncertainty (e.g. confidence intervals)
☒	☐	For null hypothesis testing, the test statistic (e.g. *F*, *t*, *r*) with confidence intervals, effect sizes, degrees of freedom and *P* value noted<br>*Give P values as exact values whenever suitable.*
☒	☐	For Bayesian analysis, information on the choice of priors and Markov chain Monte Carlo settings
☒	☐	For hierarchical and complex designs, identification of the appropriate level for tests and full reporting of outcomes
☒	☐	Estimates of effect sizes (e.g. Cohen's *d*, Pearson's *r*), indicating how they were calculated

Our web collection on statistics for biologists contains articles on many of the points above.

Software and code

Policy information about availability of computer code

Data collection	Fluorescent microscopy images were taken using NIS-elements version 4.13 (Nikon).
Data analysis	Statistical analysis was performed using Prism version 10 (GraphPad). Fluorescent microscopy images and immunoblot data were analysed using ImageJ version 2.0.0. qRT-PCR data was analysed using RotorGene Q Series 2.3.5 (Qiagen).

For manuscripts utilizing custom algorithms or software that are central to the research but not yet described in published literature, software must be made available to editors and reviewers. We strongly encourage code deposition in a community repository (e.g. GitHub). See the Nature Portfolio guidelines for submitting code & software for further information.

Data

Policy information about availability of data

All manuscripts must include a data availability statement. This statement should provide the following information, where applicable:

- Accession codes, unique identifiers, or web links for publicly available datasets
- A description of any restrictions on data availability
- For clinical datasets or third party data, please ensure that the statement adheres to our policy

All data necessary to evaluate the conclusions in this paper are included in the manuscript and/or the Supplementary files.

Research involving human participants, their data, or biological material

Policy information about studies with human participants or human data. See also policy information about sex, gender (identity/presentation), and sexual orientation and race, ethnicity and racism.

Reporting on sex and gender	Not applicable
Reporting on race, ethnicity, or other socially relevant groupings	Not applicable
Population characteristics	Not applicable
Recruitment	Not applicable
Ethics oversight	Not applicable

Note that full information on the approval of the study protocol must also be provided in the manuscript.

Field-specific reporting

Please select the one below that is the best fit for your research. If you are not sure, read the appropriate sections before making your selection.

☒ Life sciences ☐ Behavioural & social sciences ☐ Ecological, evolutionary & environmental sciences

For a reference copy of the document with all sections, see nature.com/documents/nr-reporting-summary-flat.pdf

Life sciences study design

All studies must disclose on these points even when the disclosure is negative.

Sample size	No sample size calculation was performed. Mice numbers were determined by experience in animal studies. All other experiments were repeated at least 3 times independently to ensure statistical significance of the results.
Data exclusions	No data were excluded.
Replication	All experiments except Supplementary Figure S2F-G were repeated at least 3 times independently.
Randomization	Environmental conditions, including lighting, temperature, and humidity, were kept consistent for all animals, and the cages were randomly positioned within the rack. Genetically modified cells in each experiment were derived from the same pool of parent cells.
Blinding	For the animal studies, analysis was performed blinded to the genotype of the mice. No blinding in other experiments as the same investigator performed experiment and analyzed the data.

Reporting for specific materials, systems and methods

We require information from authors about some types of materials, experimental systems and methods used in many studies. Here, indicate whether each material, system or method listed is relevant to your study. If you are not sure if a list item applies to your research, read the appropriate section before selecting a response.

Materials & experimental systems

n/a	Involved in the study	
☐	☒	Antibodies
☐	☒	Eukaryotic cell lines
☒	☐	Palaeontology and archaeology
☐	☒	Animals and other organisms
☒	☐	Clinical data
☒	☐	Dual use research of concern
☒	☐	Plants

Methods

n/a	Involved in the study	
☒	☐	ChIP-seq
☒	☐	Flow cytometry
☒	☐	MRI-based neuroimaging

Antibodies

Antibodies used	pAKTThr308 (#2965), pAKTSer473 (#4058), AKT (#4685), pAKT1Ser473 (#9018), pAKT2Ser474 (#8599), pPRAS40Thr246 (#2997), PRAS40 (#2691), pS6Ser235/236 (#4858), pS6Ser240/244 (#5364), HA (#3724), PI3K p110α (clone C73F8, #4249), PTEN (clone

	138G6, #9559) Cell Signaling Technology (Boston, MA); Ki67 (RM-9106-SO), GAPDH (#AM4300), PIPP (#PA104005) ThermoFisher Scientific (Waltham, MA); CK8 (#2031-1) Epitomics; CK14 (#ab7800) Abcam (Cambridge, MA); ACTIN (#MA5-11869) Neomarkers (ThermoFisher Scientific); HRP-conjugated secondary antibodies (Merck Millipore, Burlington, MA); fluorescently labelled secondary antibodies Molecular Probes (ThermoFisher Scientific).
Validation	All antibodies are available commercially and the validation of each primary antibody for the reactive species and applications provided by manufacturers as described.

Eukaryotic cell lines

Policy information about cell lines and Sex and Gender in Research

Cell line source(s)	T47D, MDA-MB-231, Hs578T, MCF-7, ZR-75-1, SKBR3 and BT549 human breast cancer cells and MCF-10A human mammary epithelial cells were purchased from American Type Culture Collections. SUM149PT and SUM185PE were purchased from Asterand Bioscience.
Authentication	Cell line authentication was not performed in-house but cell lines were cultured for less than 2 months.
Mycoplasma contamination	Cells were routinely tested to confirm the absence of mycoplasma contamination.
Commonly misidentified lines (See ICLAC register)	No commonly misidentified cell lines used.

Animals and other research organisms

Policy information about studies involving animals; ARRIVE guidelines recommended for reporting animal research, and Sex and Gender in Research

Laboratory animals	Female C57Bl/6 mice (wild-type, Pipp-/-, Pten+/-, Pipp-/-;Pten+/-) generated as part of this study were used. Mice were aged up to ~7 months. Mice were group housed where possible with a 12-12h light-dark cycle, 22-24°C and were fed a commercial diet ad lib.
Wild animals	The study did not involve wild animals.
Reporting on sex	Only female mice were studied to determine the relative contribution of Pipp and Pten in mammary tumorigenesis.
Field-collected samples	The study did not involve samples collected from the field.
Ethics oversight	All procedures involving mice were conducted in accordance with National Health and Medical Research Council (NHMRC) regulations on the use and care of experimental animals. The study protocol was approved by the Monash University Animal Ethics Committee (Project Numbers MARP/2013/108, MARP/13267, MARP/28260).

Note that full information on the approval of the study protocol must also be provided in the manuscript.

Plants

Seed stocks	Not applicable
Novel plant genotypes	Not applicable
Authentication	Not applicable
